# The Human Cytomegalovirus U_L_38 protein drives mTOR-independent metabolic flux reprogramming by inhibiting TSC2

**DOI:** 10.1371/journal.ppat.1007569

**Published:** 2019-01-24

**Authors:** Irene Rodríguez-Sánchez, Xenia L. Schafer, Morgan Monaghan, Joshua Munger

**Affiliations:** 1 Department of Microbiology and Immunology, School of Medicine and Dentistry, University of Rochester, Rochester, New York, United States of America; 2 Department of Biochemistry and Biophysics, School of Medicine and Dentistry, University of Rochester, Rochester, New York, United States of America; University of North Carolina at Chapel Hill, UNITED STATES

## Abstract

Human Cytomegalovirus (HCMV) infection induces several metabolic activities that are essential for viral replication. Despite the important role that this metabolic modulation plays during infection, the viral mechanisms involved are largely unclear. We find that the HCMV U_L_38 protein is responsible for many aspects of HCMV-mediated metabolic activation, with U_L_38 being necessary and sufficient to drive glycolytic activation and induce the catabolism of specific amino acids. U_L_38’s metabolic reprogramming role is dependent on its interaction with TSC2, a tumor suppressor that inhibits mTOR signaling. Further, shRNA-mediated knockdown of TSC2 recapitulates the metabolic phenotypes associated with U_L_38 expression. Notably, we find that in many cases the metabolic flux activation associated with U_L_38 expression is largely independent of mTOR activity, as broad spectrum mTOR inhibition does not impact U_L_38-mediated induction of glycolysis, glutamine consumption, or the secretion of proline or alanine. In contrast, the induction of metabolite concentrations observed with U_L_38 expression are largely dependent on active mTOR. Collectively, our results indicate that the HCMV U_L_38 protein induces a pro-viral metabolic environment via inhibition of TSC2.

## Author summary

Viruses are parasites that usurp the energy and molecular building blocks of their hosts to support their replication. In the past few years, numerous studies have shown that a wide variety of viruses induce cellular metabolic activities that are essential for successful infection. However, the viral mechanisms responsible for these metabolic alterations have remained unclear. Here, we find that the Human Cytomegalovirus (HCMV) U_L_38 gene is responsible for inducing many of the metabolic activities that are critical for successful HCMV infection. HCMV is a herpes virus that causes severe disease in newborns, as well as in those with weakened immune systems including transplant recipients and patients with common blood-based cancers. Our work shows that the U_L_38 protein drives cells to substantially increase the consumption of glucose and specific amino acids, which provide the energy and building blocks necessary to create new viral particles. Mechanistically, we find that U_L_38 triggers these metabolic changes through inhibition of a cellular tumor suppressor protein, TSC2. Collectively, our data provide substantial insight into how a viral pathogen reprograms cellular metabolism to support infection.

## Introduction

Viruses depend on cellular energy and macromolecules to support their replication. Several studies have identified specific virally-induced metabolic activities that are important for the production of viral progeny [1–9]. Further, many successful anti-viral treatments target virally-induced metabolic activities, e.g., those that target aberrant nucleotide metabolism during viral infection [10, 11]. Despite these successes, very little is known regarding the mechanisms through which viruses manipulate cellular metabolic activity. Given their importance to viral infection, identification of these mechanisms could provide novel targets for therapeutic intervention.

Human Cytomegalovirus (HCMV) is a widespread opportunistic pathogen that causes severe disease in neonates and immunosuppressed patients, such as cancer patients undergoing immunosuppressive treatment, transplant recipients and HIV positive patients [12]. HCMV infection is also associated with increased incidence and mortality of cardiovascular disease [13–15]. HCMV is a betaherpes virus with a double-stranded DNA genome of ∼240 kb that encodes for over 200 open reading frames (ORF)[12]. We and others have previously found that HCMV infection induces dramatic changes to the host cell metabolic network. These changes include the induction of central carbon metabolism, including glycolysis [1–3, 16, 17], glutaminolysis [18], tricarboxylic acid (TCA) cycle [1, 2], fatty acid biosynthesis [1, 5] and pyrimidine biosynthesis [2, 4]. However, HCMV’s impact on amino acid metabolism is much less clear. Further, the viral mechanisms responsible for metabolic manipulations are largely unknown, an important consideration given that inhibition of these metabolic changes attenuates HCMV infection [1–5, 16].

Here, we find that HCMV targets many aspects of amino acid metabolism, and that the HCMV U_L_38 protein is necessary and sufficient to drive many features of the HCMV-induced metabolic program. U_L_38 is an HCMV immediate early gene, conserved among beta-herpesviruses that is important for viral replication [19, 20], and has been found to induce mTORC1 activation [21, 22]. Our data suggest that U_L_38 reprograms cellular metabolic activities through its interaction with the tuberous sclerosis complex 2 protein (TSC2). TSC2 is a negative regulator of mTORC1 activity, but we find that U_L_38-mediated activation of various metabolic fluxes is largely independent of mTOR. Collectively, we propose that the HCMV U_L_38 protein is an important metabolic regulator that induces metabolic reprogramming through its inhibition of TSC2 but is largely independent of mTOR.

## Results

### HCMV infection reprograms cellular amino acid metabolism

**Fig 1:**
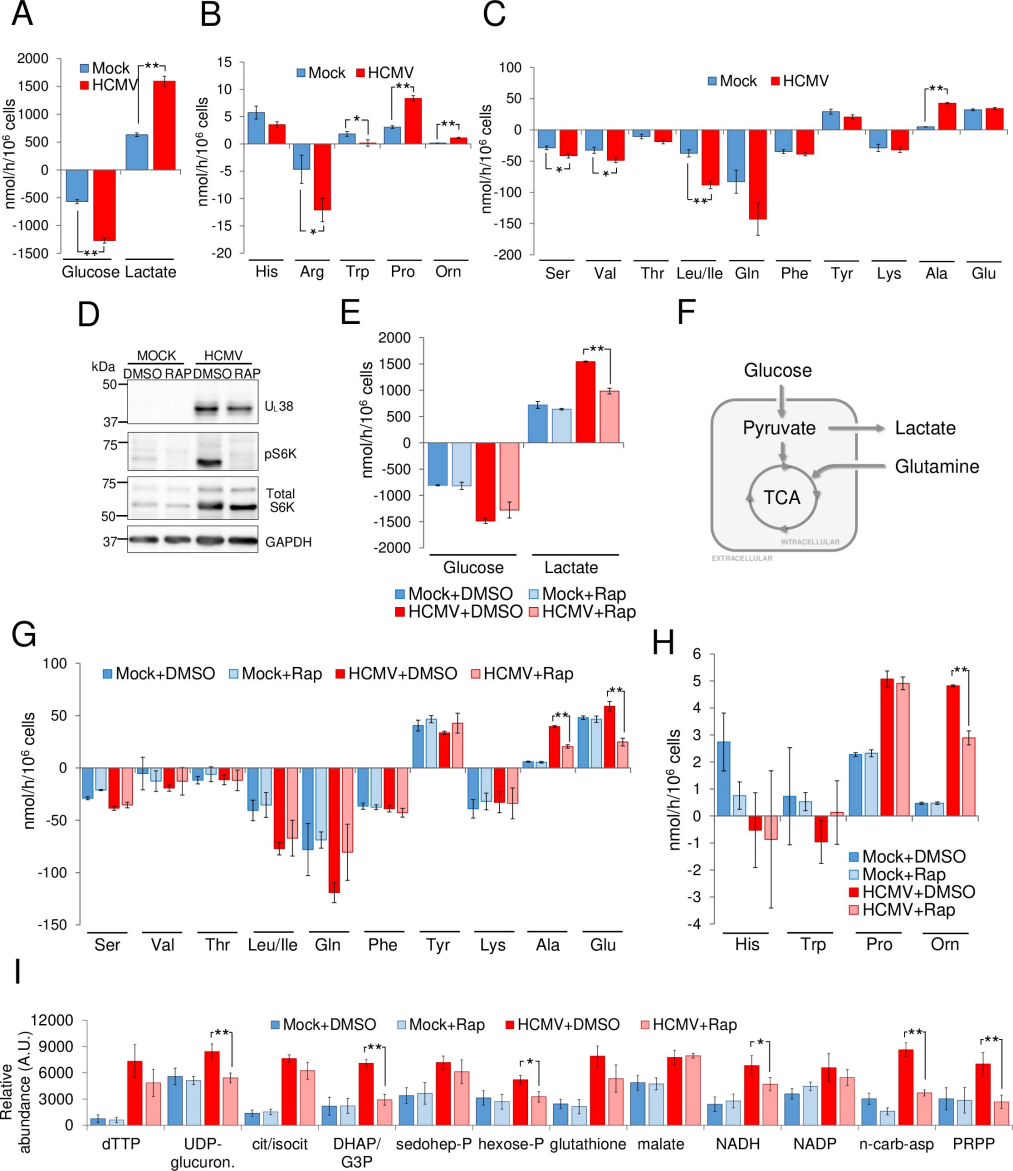
HCMV-induced metabolic reprogramming of amino acid metabolism. (A- C) MRC5 cells were mock or HCMV-infected (MOI = 3). At 36hpi, cellular medium was renewed, harvested 24h later (60hpi), and analyzed for changes in metabolite levels. Values are means ± SE (n = 6). (D, E, G & H) MRC5 cells were infected as in (A-C). At 36hpi, fresh medium containing DMSO (+DMSO) or 100 nm of rapamycin (+Rap) was added to the plates and conditioned medium and cells were harvested after 24h (60hpi). (D) Western blot analysis of drug treated mock or HCMV-infected cells (E, G & H) Changes in metabolic intermediates present in the conditioned medium were measured. Values are means ± SE (n = 3) (*p<0.05, **p<0.01). (F) Schematic of central carbon metabolism. (I) MRC5 cells were mock-infected (Mock) or infected with HCMV (HCMV) (MOI = 3) and 24h after, fresh medium containing DMSO (+DMSO) or 100 nm of rapamycin (+Rap) was added. At 48hpi cells were quenched and extracted. Absolute intracellular metabolite concentrations were determined by LC-MS/MS and normalized to protein levels. Values are means ± SE (n = 4). (*p<0.05, **p<0.01).

As previously reported, HCMV infection increases glycolysis, inducing both glucose uptake and lactate secretion (Fig 1A) [1]. Much less is known regarding how HCMV infection affects cellular amino acid dynamics. To explore this issue, we measured how HCMV infection affected the intake and secretion of amino acids in the media. HCMV infection broadly increased the consumption of several amino acids, with the consumption of leucine/isoleucine and arginine increasing the most (Fig 1B and 1C). In contrast, infection did not detectably impact the consumption of others, such as lysine and phenylalanine (Fig 1C). HCMV infection also increased the excretion of several amino acids, most notably alanine, proline and ornithine (Fig 1B and 1C). These results demonstrate that HCMV infection modulates the metabolic dynamics of several amino acids to varying extents.

The mammalian target of rapamycin complex 1 (mTORC1) coordinates cell growth, proliferation and metabolism by controlling the balance between anabolic and catabolic processes in response to environmental cues, such as nutrients or growth factors [23, 24]. Previous work has demonstrated that HCMV infection activates mTORC1 and that maintenance of this activity is required for high-titer viral replication [25, 26]. mTORC1 has been shown to regulate glycolysis, glutaminolysis, fatty acid biosynthesis, and nucleotide biosynthesis [23, 24], metabolic processes previously described to be induced during HCMV infection [1, 4, 5]. To test the role that mTOR plays in HCMV-induced modulation of central carbon metabolism, we assessed the impact of rapamycin treatment, an FDA approved mTORC1 inhibitor, on amino acid levels during HCMV infection [27, 28]. As previously reported [25, 26], HCMV infection increases the phosphorylation of S6K, a canonical mTORC1 phospho-substrate (Fig 1D). Rapamycin treatment prevented this accumulation of pS6K in both mock and HCMV-infected cells, and reduced the levels of phosphorylated 4E-BP, consistent with its inhibition of mTORC1 activity (Fig 1D and S1D Fig). Rapamycin treatment appeared to block some HCMV-induced metabolic changes, while leaving others largely unaffected. For example, rapamycin had a minimal impact on HCMV-induced glucose consumption, yet reduced lactate secretion to nearly uninfected levels (Fig 1E). This suggests that in HCMV-infected cells, mTORC1 activity preferentially drives glycolytic carbon towards lactate production and away from other glycolytic branch points, e.g., the TCA cycle (see metabolic branch point at pyruvate in Fig 1F).

Rapamycin appeared to attenuate HCMV-induced glutamine consumption (Fig 1G), a TCA cycle carbon source important for HCMV replication [18], although the changes did not reach the level of statistical significance. This rapamycin-induced decrease in glutamine consumption could potentially be playing a role in the observed reduction of lactate secretion, as a reduction in glutamine carbon suppling the TCA cycle could be compensated for by directing pyruvate into the TCA cycle and away from lactate (see branch point at pyruvate in Fig 1F). Rapamycin had little impact on HCMV-induced serine or leucine/isoleucine consumption (Fig 1G) and had no impact on HCMV-induced proline secretion, yet significantly reduced ornithine secretion (Fig 1H).

HCMV infection induces the abundance of several intracellular glycolytic, TCA cycle, and nucleotide metabolites [1, 2]. To analyze the impact of mTORC1 inhibition on these metabolic changes, we utilized LC-MS/MS to profile the impact of rapamycin treatment on intracellular metabolites pools during HCMV infection (S1A Fig). Based on these data, we subsequently constructed a partial least-squares discriminant analysis-based (PLS-DA) model (S1B and S1C Fig). HCMV and mock-infected samples segregated along the top principal component (S1B Fig), with several glycolytic, TCA cycle and nucleotide metabolites contributing most to this separation (S1C Fig). Rapamycin treatment shifted the concentrations of metabolite pools closer to those of uninfected cells (S1B Fig), including reversing HCMV-induced increases in glycolytic and nucleotide biosynthetic intermediates, e.g., dihdroxyacetone-phosphate/glyceraldehyde 3-phosphate (DHAP/G3P), hexose-phosphate, N-carbamoyl-aspartate and phosphoribosyl pyrophosphate (PRPP) (Fig 1I). In contrast, other metabolic changes induced by HCMV infection were largely rapamycin insensitive, including the increases in TCA cycle pools, e.g., citrate/isocitrate and malate (Fig 1I). Collectively, our data indicate that the relationship between mTORC1 activity and virally-induced metabolic reprogramming is complex, likely reflecting the nuances associated with mTOR-mediated metabolic regulation.

### The HCMV U_L_38 protein is necessary for HCMV-induced metabolic reprogramming

**Fig 2:**
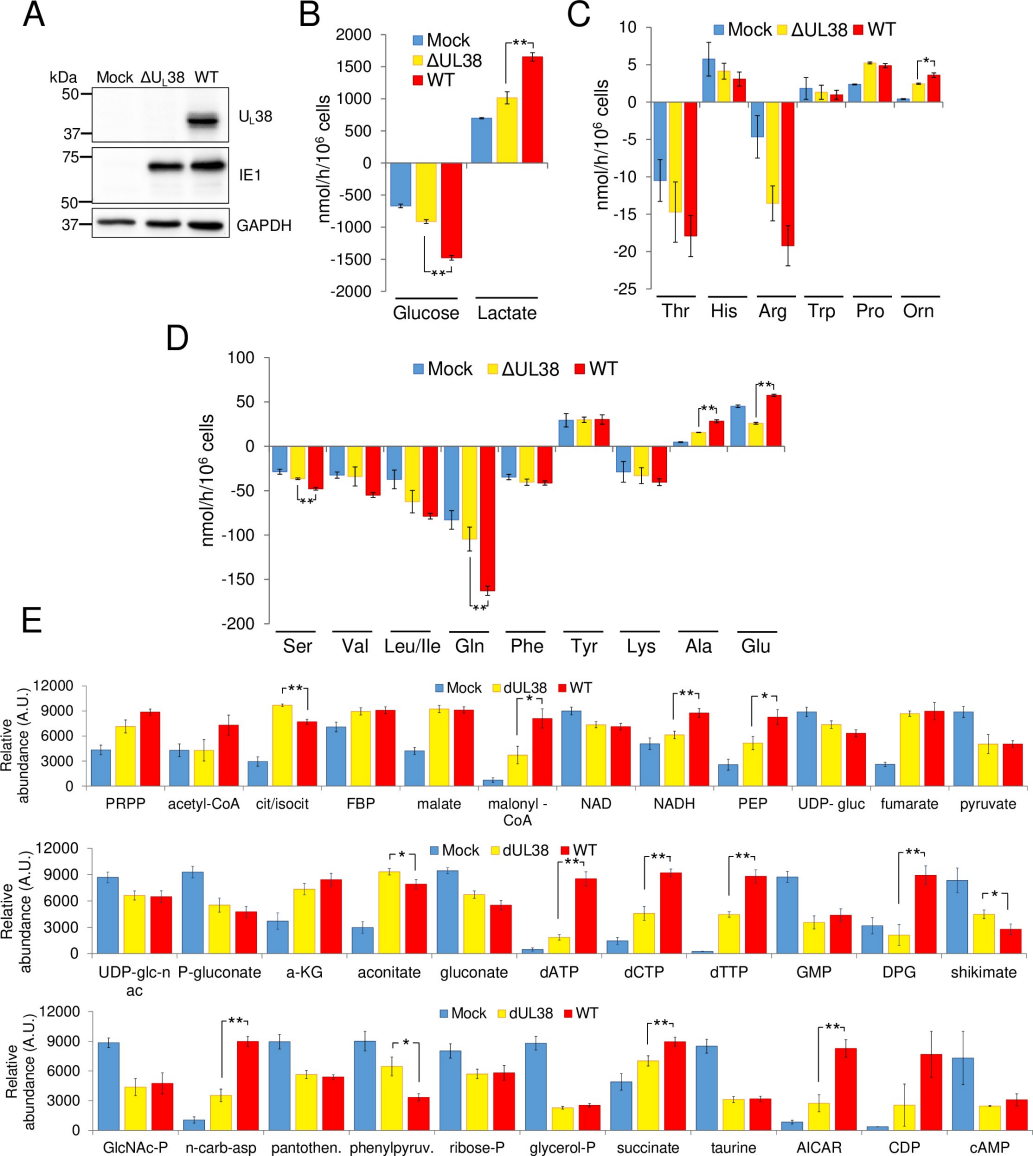
U_L_38 is important for HCMV-induced metabolic reprogramming. MRC5 cells were mock-infected, infected with a defective U_L_38 HCMV virus (ΔUL38) or infected with WT HCMV (WT) (MOI = 3). At 36hpi, medium was renewed, harvested 24h later (60hpi), and analyzed for changes in metabolite levels. (A) Western blot analysis of mock, ΔU_L_38- and HCMV-infected cells. (B-D) Changes in metabolic intermediates present in the conditioned medium were measured. Values are means ± SE (n = 4) (*p<0.05, **p<0.01). (E) MRC5 cells were mock-infected (Mock), infected with a defective U_L_38 HCMV virus (ΔUL38) or infected with WT HCMV (WT) (MOI = 3) and 24h after fresh medium was added. At 48hpi cells were quenched and extracted. Absolute intracellular metabolite concentrations were determined by LC-MS/MS and normalized to protein levels. Values are means ± SE (n = 8). (*p<0.05, **p<0.01).

The HCMV U_L_38 protein has been reported to modulate mTORC1 activation [21, 22], and since we have shown that mTORC1 is important for some metabolic changes during infection, we therefore hypothesized that the U_L_38 protein might be important for HCMV-induced metabolic reprogramming. To explore this possibility, we analyzed the impact of U_L_38 deletion on host cell metabolism during HCMV infection with a previously described U_L_38 deletion mutant (ΔU_L_38) [20]. As expected, infection with the ΔU_L_38 mutant did not accumulate U_L_38, but expressed IE1 to similar levels as WT HCMV (Fig 2A). Infection with the ΔU_L_38 mutant significantly attenuated the increases in glucose consumption and lactate secretion observed during WT HCMV infection (Fig 2B). Additionally, deletion of U_L_38 inhibited HCMV-mediated induction of serine and glutamine consumption, as well as ornithine, alanine and glutamate secretion (Fig 2C and 2D). The lack of U_L_38 during infection did not impact the consumption of phenylalanine or lysine, nor the secretion of proline or tyrosine (Fig 2C and 2D). The absence of U_L_38 also attenuated the HCMV-induced increases to several intracellular metabolite pools (S2A–S2C Fig). Both hierarchical clustering and PLS-DA-based modeling of their intracellular pool sizes suggested that mock, WT, and ΔU_L_38-infected cells largely segregate into distinct groups (S2A–S2C Fig). The metabolite pools that were significantly decreased during ΔU_L_38 infection relative to WT included N-carbamoyl-asparate, the product of the rate-determining step of pyrimidine biosynthesis, as well as pyrimidine end-products including, CDP and dTTP (Fig 2E), the production of which we have previously found to be important for HCMV infection [4]. Deletion of U_L_38 also decreased the glycolytically related metabolites NADH and phosphoenolpyruvate (PEP), as well as malonyl-CoA, the product of the rate-determining step of fatty acid biosynthesis, whose production is also important for HCMV infection [5] (Fig 2E). Collectively, the disruption of metabolic reprogramming observed during infection with the ΔU_L_38 virus indicates that the U_L_38 is important for the induction of the pro-HCMV metabolic program.

### U_L_38 is sufficient to drive metabolic reprogramming

**Fig 3:**
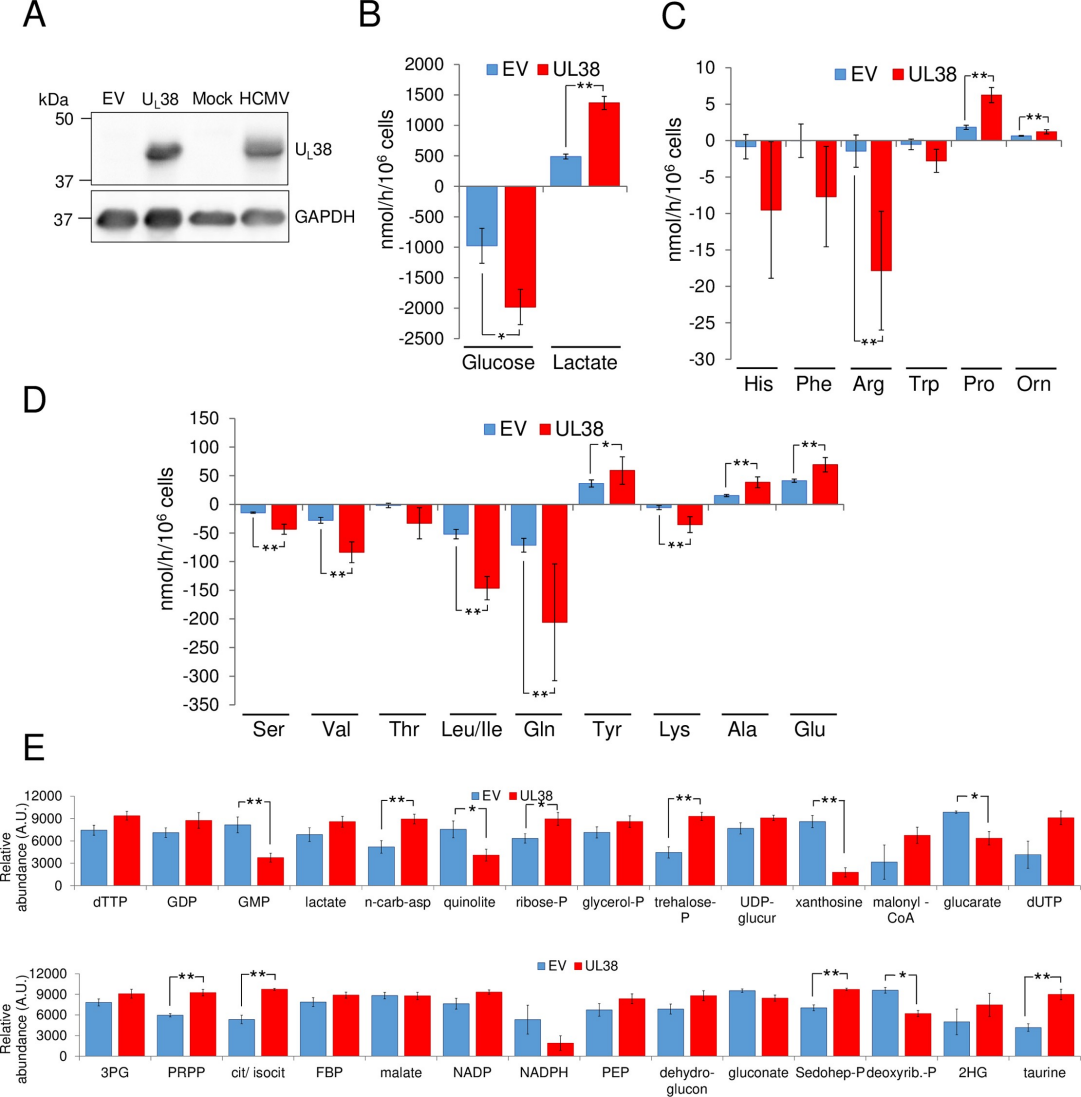
U_L_38 expression is sufficient to drive metabolic activation. After 24h incubation, conditioned serum free medium from confluent MRC5 cells transduced with an empty vector or U_L_38 was harvested for analysis. (A) Western blot analysis of U_L_38 expression in EV and U_L_38 transduced cells compared to mock and HCMV infected cells (MRC5 cells infected at MOI = 3 and harvested at 53hpi). (B-D) Changes in metabolic intermediates present in the conditioned medium were measured. Values are means ± SE (n = 3). (*p<0.05, **p<0.01). (E) Confluent MRC5 cells expressing an empty vector control (EV) or U_L_38 protein (UL38) were cultured in serum free media for 24h. Cells were then quenched and extracted for analysis. Absolute intracellular metabolite concentrations were determined by LC-MS/MS and normalized to protein levels. Values are means ± SE (n = 6). (*p<0.05, **p<0.01).

The U_L_38 protein is expressed at the earliest time of HCMV infection [20], and has been reported to be important for attenuating apoptosis during infection [20–22]. These findings raise the possibility that U_L_38’s contributions to metabolic reprogramming during infection could be an indirect consequence of other functions during viral infection. To determine if U_L_38 alone is sufficient to drive metabolic reprogramming, we expressed U_L_38 via lentiviral transduction (U_L_38) and found that it accumulated to approximately equivalent levels as during WT HCMV infection (Fig 3A). U_L_38 expression induced glucose consumption and lactate secretion (Fig 3B). U_L_38 expression also increased the influx of several amino acids including serine, valine, leucine/isoleucine and glutamine (Fig 3C and 3D), while also inducing the excretion of proline, alanine, ornithine and glutamate (Fig 3C and 3D). Expression of U_L_38 also induced increases to several intracellular metabolic pools, including citrate/isocitrate, and several key nucleotide biosynthetic intermediates such as N-carbamoyl-asparate and PRPP (Fig 3E and S3A Fig). These data suggest that U_L_38 is sufficient to drive many of the metabolic changes associated with HCMV infection in the absence of other HCMV proteins.

### U_L_38-mediated reprogramming of core central carbon metabolic fluxes is mTOR independent

**Fig 4:**
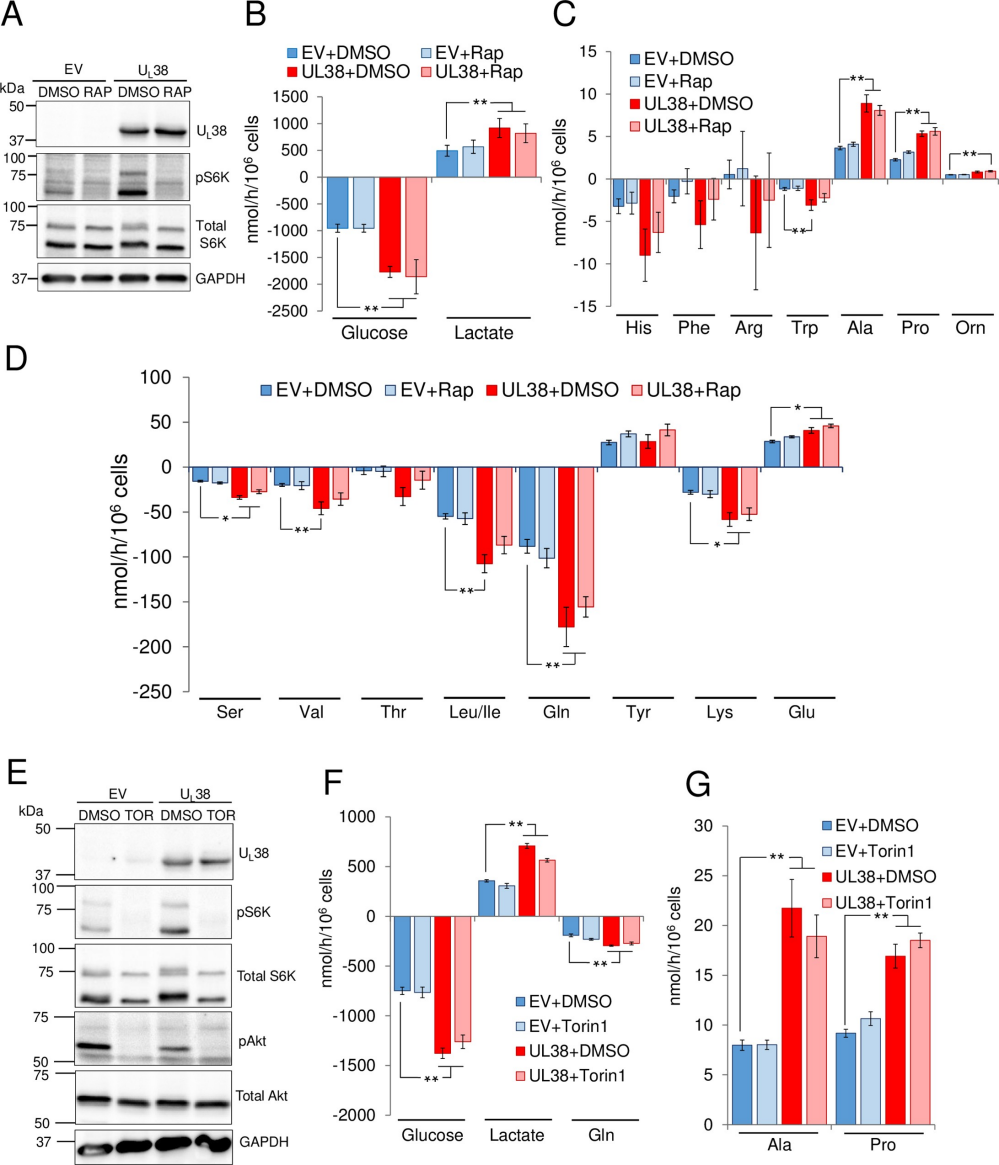
U_L_38-induced metabolic flux activation is mTOR independent. After 24h incubation, conditioned serum free medium from confluent MRC5 cells transduced with an empty vector or U_L_38 was harvested for analysis. Media contained DMSO (+DMSO), 100 nm of rapamycin (+Rap) or 250nM of Torin-1 (TOR or +Torin1) as indicated. (A, E) Western blot analysis of treated EV and U_L_38 cells. (B, C, D, F & G) Changes in metabolic intermediates present in the conditioned medium were measured. Values are means ± SE (B-D n = 9, F-G n = 8). (*p<0.05, **p<0.01).

Given that the U_L_38 protein has been reported to modulate mTORC1 activation [21, 22], and mTORC1 activity is important for some metabolic changes during HCMV infection, we sought to determine if U_L_38’s metabolic reprogramming role is dependent on mTOR activation. To that end, we treated control or U_L_38-expressing cells with rapamycin and assessed the metabolic impact. As previously reported, U_L_38 protein expression induces the activation of mTORC1 [29], as demonstrated by an increase in the abundance of phosphorylated S6K and 4EBP (Fig 4A and S4F Fig). Rapamycin treatment attenuated this activation, as indicated by the reduction in phosphorylated S6K and 4EBP levels (Fig 4A and S4F Fig). However, rapamycin treatment had little impact on U_L_38-induced glucose consumption or lactate secretion (Fig 4B). Further, rapamycin had little effect on U_L_38-mediated changes to amino acid metabolism. Alanine and proline secretion, as well as valine and lysine consumption were largely unaffected by rapamycin treatment (Fig 4C and 4D). Rapamycin did appear to reduce glutamine and leucine/isoleucine consumption to a small extent, although these changes were not statistically significant (Fig 4C and 4D). In contrast, rapamycin treatment did impact the intracellular levels of several metabolites in U_L_38-expressing cells, including several glycolytic metabolites (S4A–S4D Fig). Hierarchical clustering and PLS-DA-based modeling separated U_L_38-expressing cells from empty vector control cells regardless of rapamycin treatment (S4A–S4C Fig), suggesting that their metabolic states were distinct. However, while some metabolic pools were insensitive to rapamycin treatment, e.g., PRPP, CDP and glycerol phosphate (S4D Fig), many of the greatest U_L_38-induced increases to metabolite concentrations were reversed, including PEP, 3PG, G3P/DHAP, and malate, among others (S4D Fig). To further explore the role of mTOR in U_L_38-mediated metabolic reprogramming, we examined the impact of torin-1 treatment, which is an mTOR inhibitor that blocks both the mTORC1 and mTORC2 complexes [30]. As expected, torin-1 treatment blocked the phosphorylation of S6K, AKT and 4EBP (Fig 4E and S4F Fig). Upon torin-1 treatment, U_L_38 still induced glucose and glutamine consumption, as well as lactate, alanine and proline secretion (Fig 4F and 4G). Torin-1 treatment did reduce the U_L_38-associated increased consumption of a few amino acids, e.g., phenylalanine and arginine, but the metabolism of many were not affected, e.g., threonine, valine, glutamate, and ornithine (S4E Fig). Collectively, these data indicate that U_L_38-mediated activation of key central carbon fluxes is largely independent of mTOR activity. Further, the observation that rapamycin did not significantly impact U_L_38-induced changes to glycolysis and amino acid consumption (Fig 4B–4D), but attenuated increased metabolite concentrations, highlights that metabolite concentrations and metabolic molecular fluxes can be independently regulated. Additionally, in the context of U_L_38-mediated metabolic activation, these results suggest that mTOR is playing a larger role in the increased metabolite concentrations as compared to the increased molecular metabolic fluxes.

### A mutant U_L_38 allele (T23A/Q24A) with reduced TSC2 interaction fails to activate central carbon metabolic fluxes

**Fig 5:**
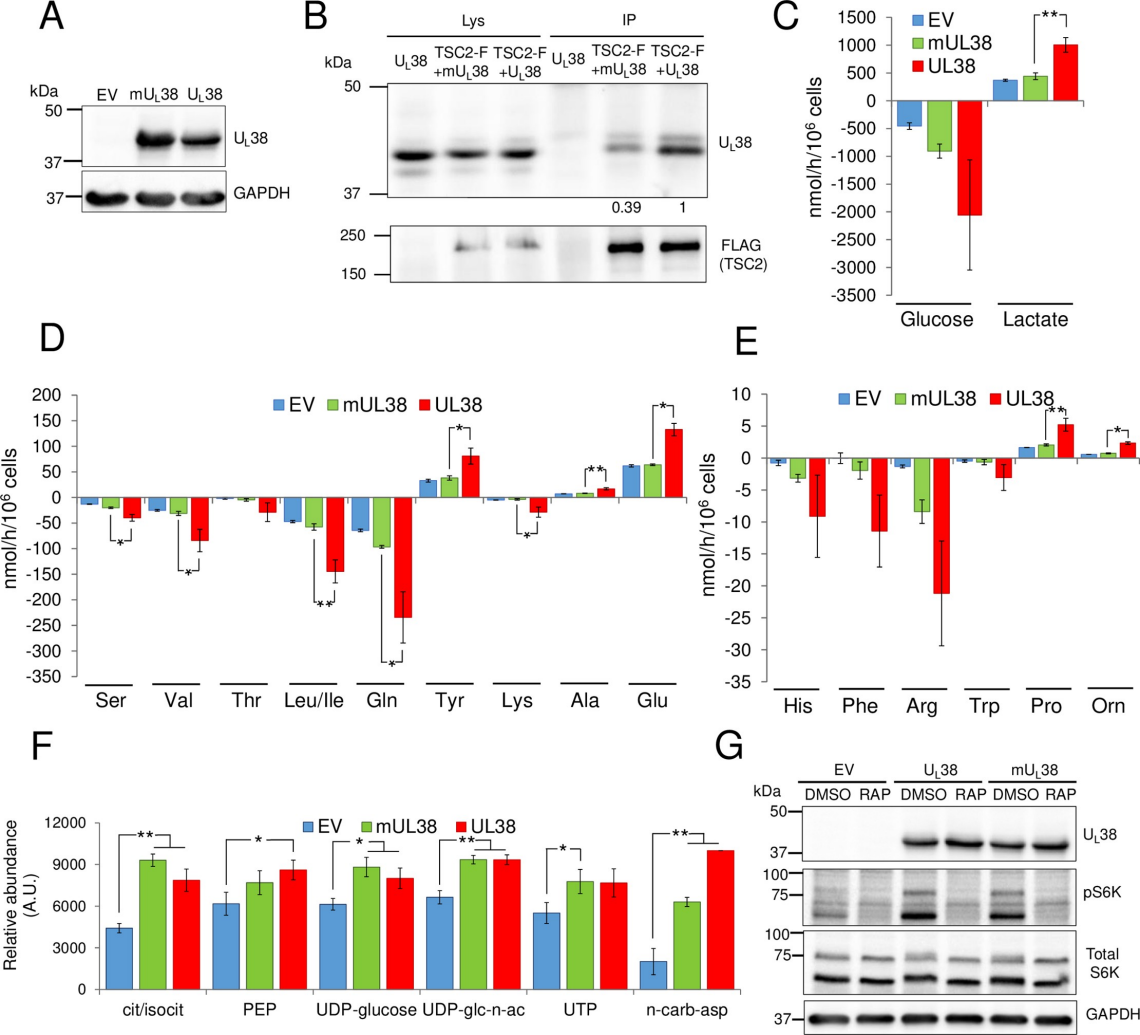
A mutant U_L_38 allele (T23A/Q24A) that shows reduced binding to TSC2 fails to activate metabolic flux. (A, C, D & E) Confluent MRC5 cells expressing an empty vector control, mutant U_L_38 T23A/Q24A (mUL38) or WT U_L_38 (UL38) were cultured in serum free media for 24h, after which conditioned medium and cells were harvested for analysis. (A) Western blot analysis of EV, mU_L_38 and U_L_38 cells. (B) 293T cells were transfected with expressing vectors for U_L_38, mU_L_38 or FLAG-TSC2 (TSC2-F) proteins, harvested 48h later and immunoprecipitated with a Flag-specific antibody. Lysate (Lys) represents 10% of the IP input. Protein band intensities are shown relative to the FLAG-TSC2+U_L_38 value. (C-E) Changes in metabolic intermediates present in the conditioned medium were measured. Values are means ± SE (n = 3). (F) Confluent MRC5 cells expressing EV, mU_L_38 or U_L_38 protein were cultured in serum free media for 24h. Cells were then quenched and extracted for LC-MS/MS analysis. Values are means ± SE (n = 9, except n-carb-asp, where n = 3). (*p<0.05, **p<0.01). (G) Confluent MRC5 cells expressing EV, mU_L_38 or U_L_38 protein were cultured in serum free media containing DMSO (DMSO) or 100 nm of rapamycin (RAP) for 24h and harvested for western blot analysis.

Previously, U_L_38 was found to bind and inhibit TSC2 [21, 22], a tumor suppressor that inhibits mTORC1 [31]. TSC2, in conjunction with TSC1, is a GTPase activating protein (GAP) for the Rheb (Ras homolog enriched in brain) GTPase [23, 24]. GTP-bound Rheb directly activates mTORC1, thus TSC2’s GAP activity inhibits mTORC1 [23, 24]. With respect to U_L_38-mediated inhibition of TSC2, previous work identified a TQ motif at amino acid residues 23 and 24 to be important for its interaction with TSC2, yet dispensable for maintaining mTORC1 activity [22]. We assessed the effects of these mutations on U_L_38-mediated metabolic modulation. Cells expressing wildtype or mutant U_L_38 exhibited similar amounts of U_L_38 protein expression (Fig 5A), and further, as previously described, wildtype U_L_38 protein interacts with TSC2 (Fig 5B), and this interaction is significantly reduced by the T23A/Q24A substitutions in U_L_38 (Fig 5B). We next tested how this mutation affected U_L_38’s metabolic reprogramming ability. Expression of U_L_38_T23A/Q24A_ (mU_L_38) failed to induce many of the metabolic phenotypes associated with wildtype U_L_38 (Fig 5C–5E). Transduction with mU_L_38 failed to activate glycolysis (Fig 5C) and did not induce U_L_38-mediated changes to amino acid consumption and secretion (Fig 5D and 5E), highlighting that the mutations that significantly reduce TSC2 interaction also strongly attenuate U_L_38’s ability to activate central carbon metabolic flux.

In contrast to the impact on glycolytic and amino acid fluxes, cells expressing mU_L_38 still exhibited increased levels of several intracellular metabolites, including central carbon metabolites, such as PEP and citrate/isocitrate, various UDP-sugar intermediates including UDP-glucose and UDP-N-acetylglucosamine, and core pyrimidine metabolites, such as N-carbamoyl-asparate and UTP (Fig 5F and S5 Fig). Further highlighting the similarity in metabolite abundances between WT U_L_38 and mU_L_38 expressing cells, there was extensive overlap between these cells with respect to hierarchical clustering and a PLS-DA model of metabolite concentrations (S5A–S5C Fig). This disconnect between metabolic flux and metabolite concentrations was observed earlier with rapamycin treatment of U_L_38-expressing cells (Fig 4 and S4 Fig), i.e. rapamycin treatment did not substantially change U_L_38-induced molecular flux rates, but did reduce intracellular metabolite pools (Fig 4 and S4 Fig). The current mU_L_38 results underscore the importance of mTORC1 in increasing metabolite pool sizes, as mutant U_L_38 expression maintains mTORC1 activation as analyzed by S6K phosphorylation (Fig 5G). Given that mU_L_38 has been demonstrated to maintain mTORC1 activation [22], our collective data suggest that mTORC1 activity is not required for U_L_38-mediated induction of metabolic flux, e.g., consumption of glucose or specific amino acids, but is important for increasing the concentrations of specific metabolite pools. In total, our results suggest that the U_L_38 TQ motif, which is important for TSC2 binding, is necessary for metabolic flux remodeling, but does not affect the mTORC1-mediated increases to specific metabolic pools.

### TSC2 knock-down phenocopies U_L_38-mediated metabolic activation

**Fig 6:**
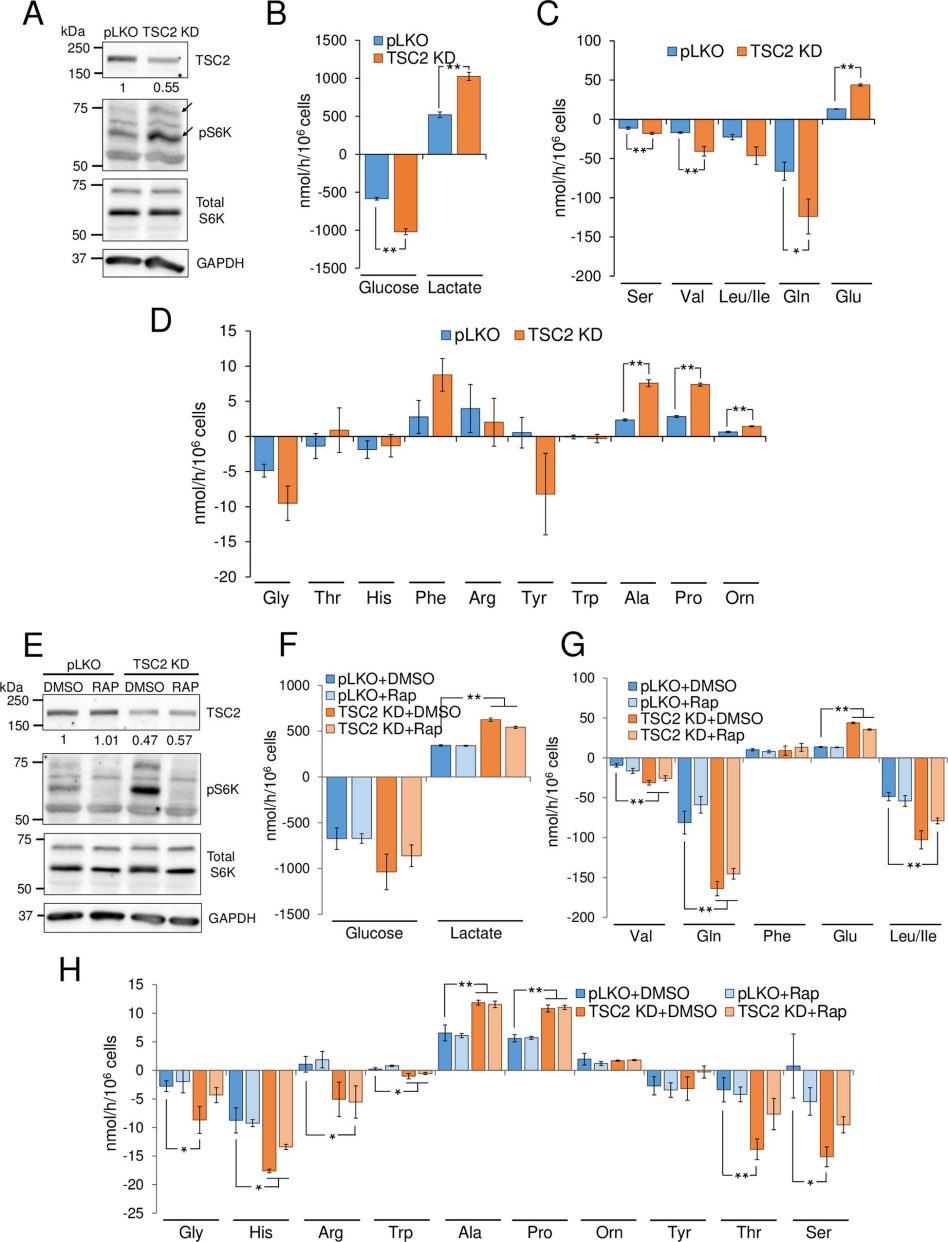
TSC2 knock-down induces activation of metabolic fluxes independent of mTORC1. (A-D) HFF cells were transduced with control (pLKO) or TSC2-specific shRNA (TSC2 KD) -expressing lentiviruses and selected. Confluent cells were cultured in serum free media for 24h, at which time the conditioned medium and cells were harvested for analysis. (A) Western blot analysis of pLKO and TSC2 KD cells. Protein band intensities are shown relative to the pLKO control value. Arrows indicate both p70 and p85 isoform of S6K (B-D). Changes in metabolic intermediates present in the conditioned medium were measured. Values are means ± SE (n = 4). (E-H) Confluent HFF cells expressing pLKO or TSC2-specific shRNA were cultured in serum free media containing DMSO (+DMSO) or 100 nm of rapamycin (+Rap) for 24h, at which time the conditioned medium and cells were harvested for analysis. (E) Western blot analysis of pLKO and TSC2 KD drug treated cells. Protein band intensities are shown relative to the pLKO+DMSO control value. (F-H) Changes in metabolic intermediates present in the conditioned medium were measured by LC-MS/MS. Values are means ± SE (n = 4). (*p<0.05, **p<0.01).

Our results suggest that U_L_38’s role in metabolic activation may be dependent on its inhibition of TSC2. This would suggest that TSC2 knockdown should result in similar metabolic phenotypes as U_L_38 expression. To test this prediction, we measured the metabolic impact of targeting TSC2 with shRNA. Lentiviral-delivered TSC2-specfic shRNA resulted in ~50% reduction in TSC2 protein abundance relative to vector control cells (Fig 6A). TSC2 knockdown also increased the accumulation of phosphorylated S6K, indicative of active mTORC1 (Fig 6A). Similar to U_L_38 expression, knockdown of TSC2 substantially increased glycolysis and lactate secretion (Fig 6B). Also analogous to expression of U_L_38, TSC2 knockdown increased glutamine, serine and valine consumption, and elevated the secretion of alanine, proline and glutamate (Fig 6C and 6D). Further, knockdown of TSC2 also induced changes to several intracellular metabolic pools, including glycolytic metabolites, UDP-sugars and nucleotide intermediates/end products such as G3P/DHAP, PEP, UDP-glucose, ADP, NADH and NADPH (S6A and S6B Fig). These results are consistent with U_L_38 modulating cellular metabolism via inhibition of TSC2.

Given that U_L_38-mediated activation of glycolytic and amino acid fluxes is rapamycin insensitive, if U_L_38 is mediating metabolic remodeling via inhibition of TSC2, we hypothesized that the metabolic flux remodeling associated with TSC2 knockdown should also be rapamycin insensitive. We assessed the effect of rapamycin treatment on the metabolic impact of TSC knockdown to test this prediction. As hypothesized, rapamycin treatment inhibited mTORC1 as demonstrated by the depletion of phosphorylated S6K (Fig 6E). Rapamycin treatment had little effect on the TSC2-knockdown-mediated induction of glucose and glutamine consumption or the excretion of lactate, alanine or glutamate (Fig 6F–6H). These results largely mirror the observations that U_L_38-mediated remodeling of many metabolic fluxes are TSC2 dependent but mTORC1 independent.

## Discussion

Viruses are obligate parasites that depend on cellular metabolic resources for their replication. Increasingly, viruses are being found to induce specific metabolic activities that are important for infection [5, 16, 32–35]. However, the mechanisms through which viruses modulate host cell metabolism have largely remained a mystery. Here we show that the HCMV U_L_38 protein is a key virally-encoded metabolic regulator. We find that U_L_38 expression is necessary and sufficient to drive multiple aspects of HCMV-mediated metabolic reprogramming, including activation of glycolytic and amino acid catabolic fluxes, activities that have been previously shown to be critical for high-titer HCMV infection [16, 18, 36]. Given the viral dependence on these metabolic activities, the mechanisms responsible may represent therapeutic vulnerabilities that could be exploited to attenuate infection.

**Fig 7:**
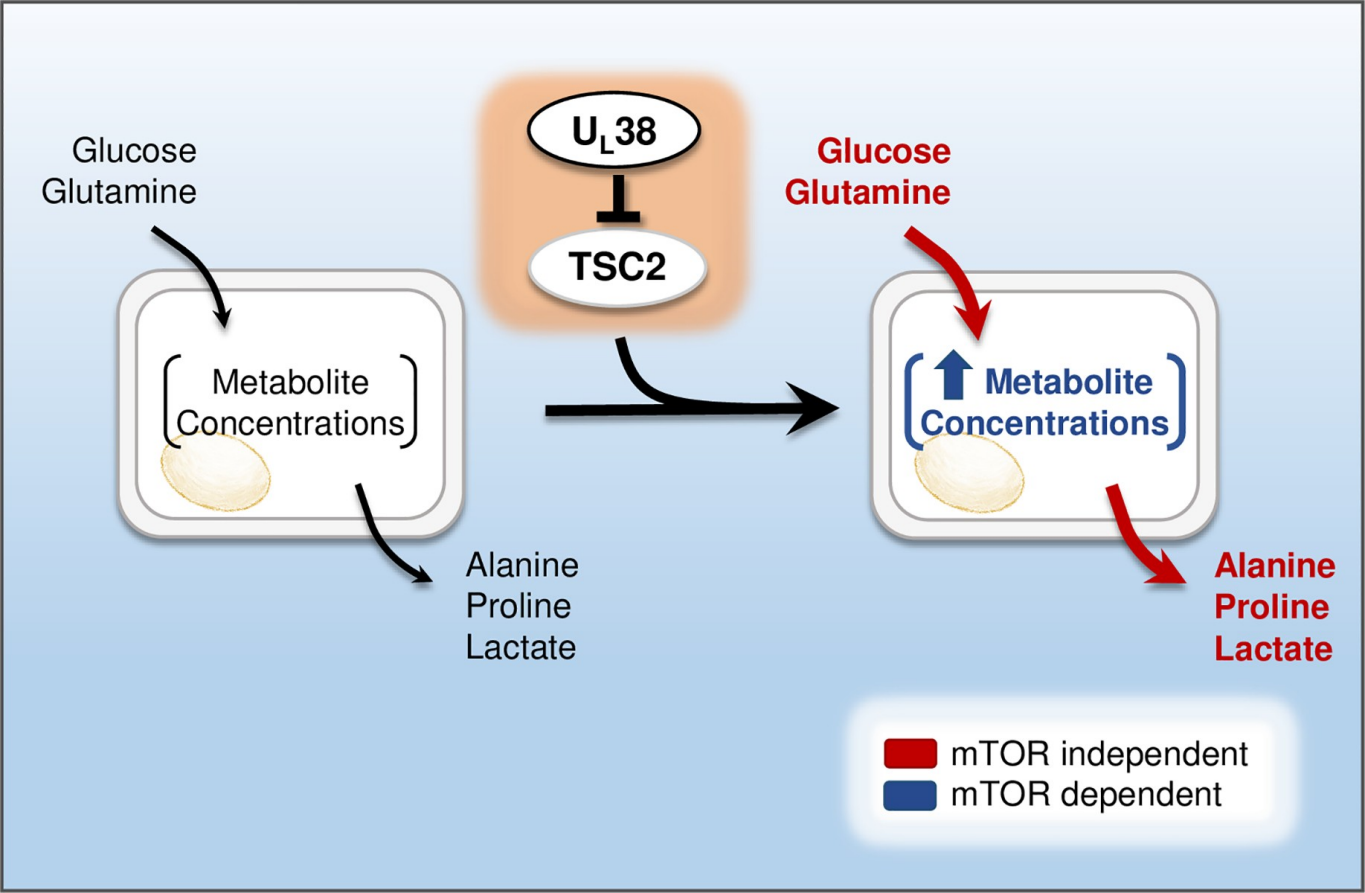
Model of U_L_38-mediated metabolic remodeling. The HCMV U_L_38 protein interacts with and inhibits TSC2, a key mTOR regulator. U_L_38 expression or TSC2 knock-down activates glycolytic and specific amino acid turnover rates, and increases metabolite concentrations. The induction of specific metabolic fluxes, e.g., glucose and glutamine consumption, and lactate, alanine and proline secretion are independent of mTOR activation, whereas the increases in metabolite pools sizes are largely mTOR dependent.

We find that the HCMV U_L_38 protein is necessary for many HCMV-induced metabolic alterations, e.g., induction of glucose and glutamine consumption as well as lactate secretion (Figs 2 and 7). Further, expression of U_L_38 is sufficient to drive many of these activities, including glucose consumption and lactate secretion, and the consumption and secretion of a number of different amino acids (Figs 3 and 7). While there was extensive overlap between the metabolic phenotypes induced by HCMV infection and those induced by U_L_38 expression, they were not identical. Several metabolic activities were induced by U_L_38 expression but not impacted by HCMV infection. For example, U_L_38 expression induced lysine consumption and tyrosine secretion, but these fluxes where not affected in the context of HCMV infection. We speculate that these changes may reflect the anabolic differences between HCMV-infected cells and uninfected cells expressing U_L_38. Specifically, virally directed biosynthetic activities such as viral protein synthesis, viral DNA replication, and envelope biogenesis, likely impact the requirements for specific amino acids, and thereby impact nutrient uptake and waste excretion.

Our results also highlight novel metabolic activities induced by HCMV infection. For example, we find that HCMV induces the secretion of ornithine, an arginine and polyamine biosynthetic intermediate, as well as proline (Fig 1). These increases were largely independent of the presence of U_L_38 (Fig 2C), suggesting that other viral factors are responsible for driving the bulk of ornithine and proline secretion. The mechanisms involved in their activation, and how these virally-induced metabolic phenotypes contribute to infection remains to be elucidated. Additionally, it is important to note that our analysis of these metabolic changes occurred over a specific time frame of infection, 36-60hpi for analysis metabolic fluxes and 48hpi for analysis of intracellular metabolic concentrations, respectively. This time frame represents a metabolically active stage in the viral life cycle, with robust viral DNA synthesis occurring. However, the metabolic consequences of infection could be substantially different at other time points of the viral life cycle. Further, the viral requirements for specific metabolic activities could change over the course of infection.

Several U_L_38-activated metabolic fluxes were largely resistant to torin-1-mediated mTOR inhibition, e.g., glucose and glutamine consumption, as well as lactate, proline and alanine secretion (Figs 4G and 7). Other U_L_38-activated fluxes were more sensitive to mTOR inhibition, most notably arginine and phenylalanine consumption (S4E Fig), indicating that mTOR plays different roles in the regulation of these metabolic pathways. In contrast to the resistance of certain metabolic fluxes to mTOR inhibition, rapamycin treatment largely reversed most of the increases in metabolite concentrations associated with U_L_38 expression. Analogously, relative to wildtype U_L_38, expression of mU_L_38 resulted in reduced metabolic fluxes, but maintained mTORC1 activity, as measured by S6K phosphorylation, and largely increased metabolite pool concentrations (Fig 5 and S5 Fig). These data indicate that metabolite concentrations and molecular flux rates can be independently regulated. Further, they suggest that in the current context, mTOR has a nuanced regulatory role in mediating metabolite concentrations and flux rates, the specific mechanisms of which require further elucidation. Given the generalized importance of metabolic regulation in a number of disease pathologies, e.g., cancer formation and metabolic syndrome, further elucidation of the mechanisms of metabolic flux control should be a high priority.

In contrast to certain core metabolic fluxes, rapamycin treatment completely reversed the HCMV-induced changes to N-carbamoyl-aspartate and PRPP (Fig 1I), key pyrimidine biosynthetic intermediates. Similar decreases in pyrimidine biosynthetic intermediates were observed in rapamycin treated U_L_38-expressing cells (S4 Fig). These results likely reflect the described roles that mTORC1 and S6K play in regulating pyrimidine metabolism [37]. Indeed, treatment with rapamycin analogs has resulted in clinical benefits with respect to HCMV infection [38]. Given that pyrimidine metabolism is important for HCMV infection [4], a possible link between the anti-HCMV effect of rapamycin and rapamycin’s impact on HCMV-induced changes to pyrimidine metabolism is worthy of further examination.

The relative insensitivity of HCMV and U_L_38-mediated metabolic activation to mTOR inhibition was similar for several metabolic fluxes, including glucose and serine consumption, as well as proline secretion (Figs 1 and 4). However, in other cases, e.g., glutamate and lactate secretion, the HCMV-induced metabolic changes appeared to be more sensitive to mTOR inhibition relative to their induction by U_L_38 expression in isolation. We speculate that the increased sensitivity to mTOR inhibition during viral infection reflects the pleotropic effects of mTOR inhibition during the viral life cycle. Numerous HCMV gene products alter various signaling pathways including, NFκB, PI3K, and various cell cycle pathways [39–41], all of which have functional links to mTOR and metabolism [42–44]. Given the role of mTOR in translational regulation, the delicate balance of viral gene interactions with these pathways would likely be dramatically affected by mTOR inhibition. We speculate that in uninfected cells expressing U_L_38, the absence of these confounding virus-host signaling interactions likely differentially impact the metabolic response to mTOR inhibition.

Our results strongly suggest that U_L_38 mediates metabolic reprogramming via inhibition of the cellular TSC2 protein. A mutant U_L_38_T23A/Q24A_ protein, which exhibits significantly reduced TSC2 binding, does not induce the activation of central carbon fluxes (Figs 5 and 7). Further, TSC2 knockdown largely phenocopies the metabolic phenotypes associated with U_L_38 expression (Fig 6). It is possible that the U_L_38_T23A/Q24A_ mutation impacts a non-TSC2-related function of U_L_38 that is important for metabolic remodeling, and therefore, U_L_38 could be inducing metabolic remodeling through a non-TSC2 mechanism. We think this is very unlikely. For one, the U_L_38_T23A/Q24A_ allele accumulates to wildtype levels and retains several functions ascribed to wildtype U_L_38 (Fig 5A and 5G and [22]). This suggests that the U_L_38_T23A/Q24A_ is not grossly defective. Further, the extent of overlap in the metabolic phenotypes associated with U_L_38 expression and TSC2 knockdown is large and unlikely to be coincidental, e.g., induction of glycolysis, glutaminolysis, as well as the consumption and secretion of several other amino acids. Collectively, these data support the model that U_L_38’s metabolic manipulation is largely due to TSC2 inhibition.

TSC2 is a tumor suppressor and well-known inhibitor of mTORC1, which globally regulates cellular metabolism in many contexts, e.g., fluctuations in nutrient availability or in response to various signal transduction pathways [45]. Surprisingly, as noted above, U_L_38’s role in inducing many metabolic fluxes appears to be independent of activated mTOR. U_L_38-mediated activation of glycolysis, glutamine consumption, and secretion of proline and alanine were resistant to mTOR inhibition (Figs 4, 6 and 7). Our results indicating that U_L_38-mediated metabolic activation depends on its interaction with TSC2 suggests that additional mTOR-independent roles for TSC2 contribute to metabolic regulation. While the vast majority of research on TSC2 focuses on its mTOR related activities, a few manuscripts describe mTOR independent activities. For example, TSC2 has been implicated in mTOR-independent vascular endothelial growth factor (VEGF) signaling, as well as in mTOR-independent stem cell self-renewal and differentiation [46, 47]. The TSC complex has also been shown to regulate PAK2 activity independently of mTOR [48]. Aside from the aforementioned mTOR-independent TSC2 phenotypes, to our knowledge, prior to this study, there is no evidence that TSC2 can regulate metabolism independent of its effects on mTOR. Further work will elucidate how these mTOR-independent activities of the TSC complex contribute to overall cellular metabolic regulation and tumor formation. With respect to HCMV infection, TSC2 inactivation and mTOR signaling can modulate diverse signaling processes including metabolism, translation and autophagy [49], and it remains to be determined how the different facets of these two important regulatory signaling components contribute to successful HCMV infection.

The U_L_38 protein is critical for successful HCMV infection [20], and has been strongly implicated in a number of diverse cellular processes. U_L_38 was first found to block ER stress-induced apoptosis [20, 50], and was subsequently found to increase mTORC1 activity [21]. Further, U_L_38 increases the expression of fatty acid elongases that are important for infection [51]. Likely as part of its role in modulating mTORC1 activity, U_L_38 also enhances the polysome association and thereby the translational efficiency of specific mRNAs [52]. These U_L_38-associated activities could be independent from one another; there are multiple examples of viral proteins with independent functional roles (reviewed in [53]). Supporting this view, mutational analysis of U_L_38 suggests that the inhibition of cell death and mTORC1 activation are separable [29]. However, functional overlap between various U_L_38 phenotypes could still exist. Numerous links exist between cellular metabolism and both translation and apoptosis. For example, amino acids levels are actively sensed by GCN2, and if amino acid levels are insufficient, translation is inhibited [54]. Further, translational regulatory controls drive the expression of rate-determining nucleotide biosynthetic enzymes to coordinate nucleotide and protein biosynthesis [55]. Similarly, glucose is actively sensed through multiple mechanisms, that ultimately induce apoptosis if concentrations are insufficient [56, 57], and activation of glycolysis has more recently been found to actively inhibit apoptotic signaling [58, 59]. Similar functional links exist between glycolysis, glycosylation and ER stress [60, 61]. While the inhibition of TSC2 appears to be critical for U_L_38-mediated metabolic modulation, the exact mechanisms through which U_L_38 modulates apoptosis and translation still require significant elucidation.

It has become clear that viruses actively modulate metabolism to support infection (reviewed in [8]). Viral metabolic modulation could be contributing to the production of energy and biomolecular subunits necessary for virion production. Other contributions include induction of lipid metabolic enzymes critical for the organization of viral maturation compartments [33, 62] or the production of specialized virion components [51]. In addition, increasing evidence suggests that metabolic signaling is playing a deterministic role in various cell fate decisions, including modulating cell death [63], immune responses [64] and stem cell differentiation [65]. Collectively, these findings raise the possibility that viral metabolic manipulation could potentially be more complex than providing a single metabolic activity to support infection, but rather, could be responsible for inducing a broader pro-viral cellular state. In this regard, it remains to be determined whether evolutionarily divergent viruses induce similar metabolic states to support infection. If so, the mechanisms to do so would likely diverge, e.g., U_L_38 is only conserved among beta herpesviruses. Regardless, it seems very likely that host cells do not simply cede the metabolic controls to viral pathogens, but rather that these controls serve as a core host-pathogen interaction. Here, we find that the HCMV U_L_38 protein is a major viral player in this interaction, driving a large portion of the HCMV-induced metabolic program through targeting the cellular TSC2 metabolic regulator.

## Materials and methods

### Cell culture and viral infections

Human 293T cells (ATC CCRL-3216), MRC5 human fibroblasts (ATCC CCL-171), telomerase-immortalized HFF fibroblasts (HFF), telomerase-immortalized MRC5 fibroblasts and their derived recombinant cells lines (see below) were cultured in Dulbecco's modified Eagle medium (DMEM; Invitrogen) supplemented with 10% fetal bovine serum, 4.5 g/liter glucose, and 1% penicillin-streptomycin (Pen-Strep; Life Technologies) at 37°C in a 5% (vol/vol) CO_2_ atmosphere. All experiments involving MRC5 cells utilized MRC5 cells that express hTERT, with the exception of the experiments in S1 Fig, which were performed using non-hTERT expressing MRC5 cells. Before HCMV infection, MRC5 cells were grown to confluence, resulting in ∼3.2 × 10^4^ cells per cm^2^. Once confluent, medium was removed, and serum-free medium was added. Cells were maintained in serum-free medium for 24h before infection at which point they were mock infected or infected at a multiplicity of infection of 3.0 pfu/ cell. After a 2h adsorption period, the inoculum was aspirated and fresh serum-free medium was added. Conditioned medium and cells were harvested for metabolic, transcriptional, or total protein analysis at various times after the initiation of infection. Unless indicated otherwise, the strain utilized for viral infections was BADwt derived from a bacterial artificial chromosome (BAC) clone of the HCMV AD169 laboratory strain [66]. The recombinant HCMV-ΔU_L_38 BAC derived virus which lacks the entire U_L_38 allele, was courteously provided by Thomas Shenk, Princeton University (ADdlU_L_38) [20]. For counting cells, adherent cells were washed with phosphate-buffered saline (PBS), trypsinized and homogenized in supplemented DMEM medium. An aliquot of the cell suspension was mixed 1:1 with 0.4% trypan blue solution and counted using a TC10 automated cell counter (Bio-Rad), following the manufacturer's instructions. Live cell counts, i.e. trypan blue excluding cells, were used for normalizations.

### Compounds

Rapamycin (Sigma-Aldrich) and Torin-1 (ApexBio) were prepared at 100uM and 250uM respectively in dimethyl sulfoxide (DMSO). Standards for LC-MS flux analysis that were not present in DMEM include: Lactic acid (Acros Organics), L-glutamic acid (Sigma-Aldrich), L-alanine (VWR), L-ornithine (Alfa Aesar) and L-proline (Alfa Aesar), which were prepared in OmniSolv Water (MilliporeSigma) at 710 mM, 16 mM, 16 mM, 0.5mM and 8mM respectively.

### Cloning

The human telomerase (hTERT) cDNA was amplified by PCR from pWZL-Blast-Flag-HA-hTERT (Addgene plasmid 22396) using the following primers: forward primer 5′-GGAACCAATTCAGTCGACTGGGATCCCGTCCTGCTGCGCACGTG-3′ and reverse primer 5′-TTTGTACAAGAAAGCTGGGTTCTAGATCAGTCCAGGATGGTCTTGAAGTCTG-3′. hTERT cDNA was then cloned via Gibson assembly into the BamHI and XbaI sites of pLenti CMV/TO/Hygro (Addgene plasmid 17484) [67]. Wild type TB40/e UL38 allele (UL38) was amplified by PCR from the TB40/e BAC clone (EF999921.1) using the following primers: forward primer 5’- CTTTAAAGGAACCAATTCAGTCGACTGGATCATGACTACGACCACGCATAGCACCGCCGC-3’ and reverse primer 5’- AACCACTTTGTACAAGAAAGCTGGGTCTAGCTAGACCACGACCACCATCTGTACCACGTC-3’.

A TB40/e mutant UL38 allele-T23A/Q24A (mUL38) was synthetized as a 996bp gBlocks Gene Fragment (IDT) using the TB40/e UL38 sequence (EF999921.1) and mutating the sequence corresponding to the 23 and 24 amino acids [22]. This construct was amplified by PCR using the same primers described above for the wild type UL38 allele. Both U_L_38 and mU_L_38 constructs were then cloned via Gibson assembly into a BamHI and XbaI digested pLenti CMV/TO Puro plasmid (Addgene plasmid 22262). pLenti CMV/TO/Puro/empty (EV) was provided by Hartmut Land, University of Rochester.

### Lentiviral transfection and transduction

293T cells were seeded at 2 × 10^6^ cells per 10-cm dish and grown for 24h. For the generation of pseudotyped lentivirus, each 10-cm dish of 293T cells was transfected with 2.6 μg lentiviral vector, 2.4 μg PAX2, and 0.25 μg vesicular stomatitis virus G glycoprotein using the Fugene 6 reagent (Promega). Twenty-four hours later, the medium was removed and replaced with 4 ml of fresh medium. Lentivirus–containing medium was collected after an additional 24 h and filtered through a 0.45μm pore-size filter prior to transduction. The fibroblasts were transduced with lentivirus in the presence of 5 μg/ml Polybrene (Millipore Sigma) and incubated overnight. The lentivirus-containing medium was then removed and replaced with fresh DMEM. At 72 h after transduction, the cells were placed under selection with antibiotics. Cells transduced with pLenti CMV/TO/Hygro/hTERT were grown in 200 μg/ml Hygromycin B (Invitrogen) for 1 week, and the expression of hTERT was confirmed by quantitative PCR (qPCR). Cells transduced with pLenti CMV/TO/Puro/empty, pLenti CMV/TO/Puro/UL38 or pLenti CMV/TO/Puro/mUL38 were selected in 10 μg/ml Puromycin (MilliporeSigma) for 4 days. At the time of antibiotic selection of transduced cells, non-transduced control cells were also treated with Puromycin or Hygromycin as appropriate to ensure killing of non-transduced cells and ubiquitous transduction efficiencies. The expression of U_L_38 in these cells was confirmed by Western blot (WB). Empty vector or U_L_38-expressing cells were cultured in serum free media for 24 h prior to analysis.

### Immunoprecipitation

293T cells (~60% confluent) grown in 10-cm dishes were transiently transfected with pRK7-FLAG-TSC2 (Addgene plasmid 8996), CMV/TO/Puro/UL38 or pLenti CMV/TO/Puro/mUL38 using the Fugene 6 reagent (Promega) according to the manufacturer's instructions. Twenty-four hours later, the medium was removed and replaced with fresh medium. Forty-eight hours post-transfection, cells were scraped and harvested in 750 ul of RIPA buffer (Tris-HCl, 50 mM, pH 7.4; 1% Triton X-100; 0.25% Na-deoxycholate; 150 mM NaCl; 1 mM EDTA) supplemented with Pierce Protease Inhibitor tablets (PI; Thermo Scientific). Lysates were sonicated and incubated on ice for 30 min with vortexing for 5 sec every 5 min. Insoluble material was pelleted by centrifugation at 16,000 x g for 5 min at 4^o^ C. ANTI-FLAG M2 Affinity Gel (Sigma-Aldrich) in RIPA+PI buffer was added and the sample was incubated for 2h at 4^o^ C with rotation. The agarose beads were pelleted and washed 5 times with RIPA+PI buffer. Following the final wash, residual buffer was removed and the beads were resuspended in disruption buffer (see below), boiled at 100°C for 5 min, and insoluble material pelleted by spinning for 3 min at room temperature at 16,000 x g. Samples were resolved on 10% SDS-containing polyacrylamide gels, and proteins were identified by Western blot [68].

### Protein analysis

For Western blot assays [69] cells were scraped and solubilized in disruption buffer containing 50 mM Tris (pH 7.0), 2% SDS, 5% 2-mercaptoethanol, and 2.75% sucrose. The resulting extracts were sonicated, boiled for 5 min, and centrifuged at 14,000 × g for 5 min to pellet insoluble material. The extracts were then subjected to electrophoresis in an 8 or 10% SDS polyacrylamide gel and transferred to a nitrocellulose sheet. The blots were then stained with Ponceau S to ensure equivalent protein loading and transfer, blocked by incubation in 5% milk in TBST (50 mM Tris-HCl, pH 7.6, 150 mM NaCl, 0.1% Tween 20), and reacted with primary and, subsequently, secondary antibodies. Protein bands were visualized using an enhanced chemiluminescence (ECL) system (Bio-Rad) and by using the Molecular Imager Gel Doc XR+ system (Bio-Rad). For protein band quantifications, the Molecular Imager Gel Doc was used and band intensities were integrated by using ImageJ software. The antibodies used were specific for p70 S6 Kinase (S6K; Cell Signaling), phospho-p70 S6 Kinase (Thr389) (pS6K; Cell Signaling), tuberin (TSC2; Santa Cruz Biotechnology), glyceraldehyde-3-phosphate dehydrogenase (GAPDH; Cell Signaling Technology) anti-U_L_38 (8D6) [20], anti-IE1 [70] and ANTI-FLAG M2 (Sigma-Aldrich). For total protein analysis, cells were washed with PBS, scraped in 1ml of RIPA buffer supplemented with Pierce Protease Inhibitor tablets (Thermo Scientific) and vortexed. After 10 min on wet ice, lysates were centrifuged at 14,000 × g for 10 min. The protein concentration of supernatants was determined by using the Bradford assay (Bio-Rad).

### shRNA knockdown

Human TSC2 mRNA expression was targeted by using a TSC2-specific MISSION shRNA construct (#TRCN0000010454, Sigma-Aldrich) selected after a screening process in which TSC2-knockdown was assessed by qPCR and WB. For the shRNA transductions, pseudotyped lentiviruses were generated using the previously mentioned lentiviral transfection protocol using #TRCN0000010454 vector and non-target control MISSION pLKO.1-puro (SHC001; Sigma-Aldrich). HFF fibroblasts at 30% confluence were transduced with half of the filtered lentivirus-containing medium supplemented with 5ug/ml of polybrene. Cells were incubated overnight and the lentivirus-containing medium was then removed and replaced with fresh DMEM. At 72 h after transduction, the cells were placed under 10 μg/ml Puromycin selection for 4 days. The knockdown of TSC2 in these cells was confirmed by Western blot (WB) for all subsequent experiments.

### Measurement of metabolic fluxes and concentrations

For quantification of metabolic consumptions and secretions, cells were plated in 10-cm dishes. Once confluent, medium was removed and serum-free medium was added with or without chemical inhibitors as indicated. An aliquot of this virgin medium was saved to be used as t = 0 control. Cells were maintained in this serum-free medium for 24h, at which time conditioned medium was collected for glucose measurement or LC-MS/MS analysis, and cells were harvested for qPCR, WB or cell counts.

Glucose consumption rates were quantified using the HemoCue Glucose 201 System (HemoCue). A glucose standard curve was utilized for each experiment using the t = 0 virgin DMEM medium (4.5 g/liter glucose) serially diluted in PBS. Conditioned medium samples were then diluted serially 1/4 in PBS to ensure signal linearity. The glucose present in each sample was measured using the HemoCue System and normalized using the generated standard curve. To obtain consumption values, the glucose value measured for normalized virgin DMEM medium was subtracted from the result of each normalized conditioned medium value. These values were then normalized to the number of live cells counted in each plate. A negative rate indicates glucose has been consumed (less glucose in the conditioned medium than in the virgin medium).

For quantification of metabolic fluxes, serially diluted supplemented t = 0 virgin DMEM (see compounds section) and conditioned medium samples diluted 1/2 in OmniSolv Water were diluted 1/100 in 80% methanol. Samples were then centrifuged at 4°C for 5 minutes at full speed to pellet insoluble material. For amino acid quantification, 100 μl of the above methanol dilutions were derivatized with 1 μl benzyl chloroformate and 5 μl trimethylamine. The samples were then centrifuged at 4°C for 5 minutes at full speed to pellet insoluble material and subsequently analyzed by LC-MS/MS as indicated below. For lactate quantification, 100ul of the underivatized methanol dilutions were centrifuged at 4°C for 5 minutes at full speed to pellet insoluble and analyzed by LC-MS/MS as indicated below.

For quantification of intracellular metabolite concentrations, cells were plated in 10-cm dishes and once confluent, medium was removed and changed to serum-free medium supplemented with 10mM HEPES, 1% penicillin-streptomycin and chemical inhibitors as indicated. Cells were maintained in this serum-free medium for 24h, and one hour prior to metabolite extraction medium was once again changed. Medium was aspirated and 80:20 OmniSolv Methanol: OmniSolv Water (80% methanol) at −80°C was immediately added to quench metabolic activity and extract metabolites. Cells were then incubated at −80°C for 10 min. Following cell quenching, cells were scraped in the dish and kept on dry ice, and the resulting cell suspension vortexed, centrifuged at 3,000 × g for 5 min, and reextracted twice more with 80% methanol at −80°C. After pooling the three extractions, the samples were completely dried under N2 gas, dissolved in 175 μl 50:50 OmniSolv Methanol: OmniSolv Water methanol, and centrifuged at 13,000 × g for 5 min to remove debris. Samples were loaded in the LC-MS/MS for analysis as indicated below.

### LC-MS/MS analysis and normalization

Metabolites were analyzed using reverse phase chromatography with an ion-paring reagent in a Shimadzu HPLC coupled to a Thermo Quantum triple quadrupole mass spectrometer running in negative mode with selected-reaction monitoring (SRM) specific scans as previously described [4, 71]. LC-MS/MS data were then analyzed using the publicly available mzRock machine learning toolkit (http://code.google.com/p/mzrock/), which automates SRM/HPLC feature detection, grouping, signal to noise classification, and comparison to known metabolite retention times [72].

For relative quantification of intracellular metabolite levels, protein-normalized peak heights were normalized by the maximum value for a specific metabolite measured across the samples run on a given day. This normalization serves to reduce the impact of inter-day mass spectrometry variability, i.e. batch effects, while preserving relative differences between samples.

For quantification of metabolite consumption and secretion, the concentrations of control cell (either Mock or EV cells) media metabolites were estimated by comparing the extracted ion chromatograms of metabolite-specific SRM peak heights to those of metabolite standard dilution curves. Extracts of control cell media were subsequently used as standards to estimate the absolute media metabolite abundances for the other samples. Consumption and secretion rates were obtained by subtracting the concentration of virgin medium metabolites from the conditioned media metabolite concentrations. The resulting values were then normalized to the number of live cells counted for each sample. A negative rate indicates the compound has been consumed (less of that compound in the conditioned medium than in the virgin medium) and a positive rate indicates that the metabolite has been secreted into the medium.

### Statistics

Statistical analysis of the reported metabolic data were performed using JMP Statistical Analysis Software (https://www.jmp.com/). Response Screening was performed using one-way ANOVA, with False Discovery Rate (FDR) correction as described [73]. Robust Estimation, i.e. a Huber M-estimation, was employed to limit the sensitivity of outliers. Data were judged significantly different if the robust estimated FDR-corrected value p-value was <0.05. Although plotted separately, for the most accurate statistical modeling, and to increase the associated statistical power, the data comparing EV versus U_L_38 in Fig 3 and Fig 5 were combined for statistical comparisons. Statistics for all figures are available in S1 File.

Protein normalized concentration data were utilized for PLS-DA modeling and hierarchical clustering, both of which were performed using the publicly available software MetaboAnalyst 3.0 (http://www.metaboanalyst.ca) [74]. PLS-DA regression was performed using the plsr function provided by the R pls package [75]. Model classification and cross-validation were performed using the corresponding wrapper function in the R caret package [76]. Permutation testing was performed on the PLS-DA model class assignments, with 1,000 permutations, yielding a p-value less than 10^−3^. Agglomerative hierarchical clustering was performed with the hclust function in R stat package, using Euclidean distance as the similarity measure, and Ward’s linkage as the clustering algorithm.

## Supporting information

S1 Fig The impact of rapamycin treatment on HCMV-induced metabolite pools. MRC5 cells were mock-infected (Mock) or infected with HCMV (HCMV) (MOI = 3) and 24h after, fresh medium containing DMSO (+DMSO) or 100 nm of rapamycin (+Rap) was added. At 48hpi cells were quenched and extracted. Absolute intracellular metabolite concentrations were determined by LC-MS/MS and normalized to protein levels. (A) Heatmap of clustered metabolite pools. (B) Partial least-squares discriminant analysis (PLS-DA) of metabolic concentrations. (C) Loading plot for PLS-DA model. Values are means ± SE (n = 4). (*p<0.05, **p<0.01). (D) Western blot analysis of mock and HCMV-infected drug treated cells. MRC5 cells were mock or HCMV-infected (MOI = 3). At 36hpi, fresh medium containing DMSO (DMSO), 100 nm of rapamycin (Rap) or 250nM of Torin-1 (Torin1) were added to the plates and cells were harvested after 24h (60hpi).
(TIF)

S2 Fig U_L_38 protein is important for the induction of several intracellular metabolic pools during HCMV infection. MRC5 cells were mock-infected (Mock), infected with a defective U_L_38 HCMV virus (ΔUL38) or infected with WT HCMV (WT) (MOI = 3) and 24h after fresh medium was added. At 48hpi cells were quenched and extracted. Absolute intracellular metabolite concentrations were determined by LC-MS/MS and normalized to protein levels. (A) Heatmap of clustered metabolite pools. (B) Partial least-squares discriminant analysis (PLS-DA) of metabolic concentrations. (C) Loading plot for PLS-DA model. Values are means ± SE (n = 8). (*p<0.05, **p<0.01).
(TIF)

S3 Fig U_L_38 expression is sufficient to induce several intracellular metabolic pools. Confluent MRC5 cells expressing an empty vector control (EV) or U_L_38 protein (UL38) were cultured in serum free media for 24h. Cells were then quenched and extracted for analysis. Absolute intracellular metabolite concentrations were determined by LC-MS/MS and normalized to protein levels. (A) Heatmap of clustered metabolite pools. Values are means ± SE (n = 6). (*p<0.05, **p<0.01).
(TIF)

S4 Fig Impact of mTOR inhibitors on U_L_38-induced metabolic reprogramming. (A-D) Confluent MRC5 cells expressing an empty vector control (EV) or UL38 protein (UL38) were cultured in serum free media containing DMSO (+DMSO) or 100 nm of rapamycin (+Rap) for 24h. Cells were then quenched and extracted. Absolute intracellular metabolite concentrations were determined by LC-MS/MS and normalized to protein levels. (A) Heatmap of clustered metabolite pools. (B) Partial least-squares discriminant analysis (PLS-DA) of metabolic concentrations. (C) Loading plot for PLS-DA model. (D) Plotted selected metabolites. Values are means ± SE (n = 8). (E) Confluent MRC5 cells expressing EV or U_L_38 protein were cultured for 24h in serum free media containing DMSO (+DMSO) or Torin-1 (+Torin1). Conditioned medium and cells were harvested after 24h for analysis. Values are means ± SE. (n = 8) (*p<0.05, **p<0.01). (F) Western blot analysis of drug treated EV and U_L_38 cells (D = DMSO; R = Rapamycin; T = Torin1). Samples correspond to experiments described in Fig 4.
(TIF)

S5 Fig The mutant U_L_38 allele (T23A/Q24A) maintains the induction of intracellular metabolic pools. Confluent MRC5 cells expressing an empty vector control (EV), mutant UL38 T23A/Q24A (mUL38) or WT UL38 (UL38) were cultured in serum free media for 24h prior to metabolic quenching and extraction. Cellular absolute intracellular metabolite concentrations were determined by LC-MS/MS and normalized to protein levels. (A) Heatmap of clustered metabolite pools. (B) Partial least-squares discriminant analysis (PLS-DA) of metabolic concentrations. (C) Loading plot for PLS-DA model. (D) Plotted selected metabolites. Values are means ± SE (n = 9). (*p<0.05, **p<0.01).
(TIF)

S6 Fig Impact of TSC2 knockdown on cellular metabolite pool concentrations. HFF cells were transduced with control (pLKO) or TSC2-specific shRNA (TSC2 KD)-expressing lentiviruses and selected. Confluent cells were cultured in serum free media for 24h before quenching and extraction. Absolute intracellular metabolite concentrations were determined by LC-MS/MS and normalized to protein levels. (A) Heatmap of clustered metabolite pools. (B) Plotted selected metabolites. Values are means ± SE (n = 3).
(TIF)

S1 File Statistical comparisons for all experiments. (XLSX)
