# Supplementary figures and images for: The Human Cytomegalovirus UL38 protein drives mTOR-independent metabolic flux reprogramming by inhibiting TSC2
Source: PLoS Pathog. 2019 Jan 24;15(1):e1007569. doi: 10.1371/journal.ppat.1007569 (PMC6363234; doi:10.1371/journal.ppat.1007569)

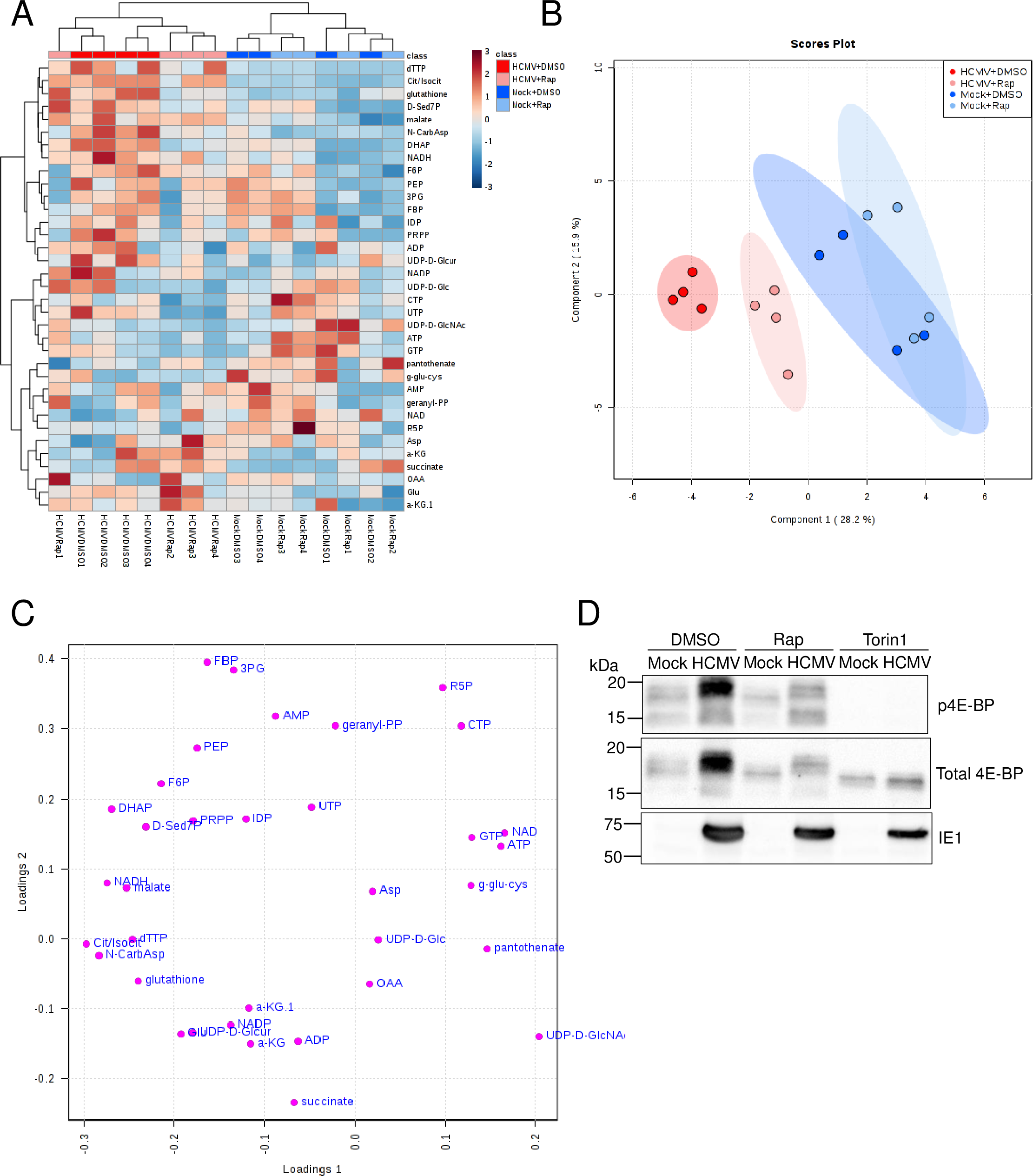

Supplement: S1 Fig — MRC5 cells were mock-infected (Mock) or infected with HCMV (HCMV) (MOI = 3) and 24h after, fresh medium containing DMSO (+DMSO) or 100 nm of rapamycin (+Rap) was added. At 48hpi cells were quenched and extracted. Absolute intracellular metabolite concentrations were determined by LC-MS/MS and normalized to protein levels. (A) Heatmap of clustered metabolite pools. (B) Partial least-squares discriminant analysis (PLS-DA) of metabolic concentrations. (C) Loading plot for PLS-DA model. Values are means ± SE (n = 4). (*p<0.05, **p<0.01). (D) Western blot analysis of mock and HCMV-infected drug treated cells. MRC5 cells were mock or HCMV-infected (MOI = 3). At 36hpi, fresh medium containing DMSO (DMSO), 100 nm of rapamycin (Rap) or 250nM of Torin-1 (Torin1) were added to the plates and cells were harvested after 24h (60hpi). (TIF) [file ppat.1007569.s001.tif]

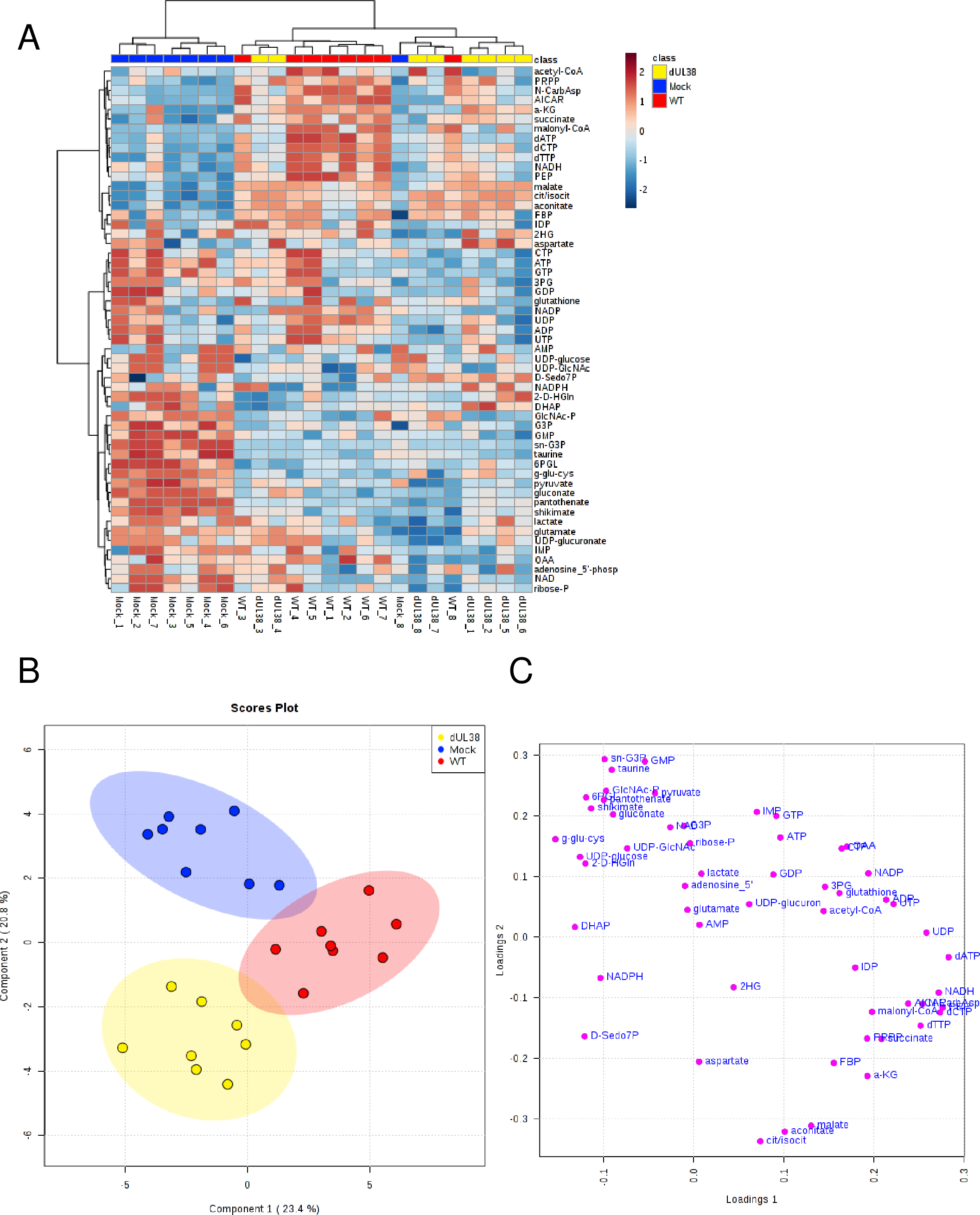

Supplement: S2 Fig — MRC5 cells were mock-infected (Mock), infected with a defective UL38 HCMV virus (ΔUL38) or infected with WT HCMV (WT) (MOI = 3) and 24h after fresh medium was added. At 48hpi cells were quenched and extracted. Absolute intracellular metabolite concentrations were determined by LC-MS/MS and normalized to protein levels. (A) Heatmap of clustered metabolite pools. (B) Partial least-squares discriminant analysis (PLS-DA) of metabolic concentrations. (C) Loading plot for PLS-DA model. Values are means ± SE (n = 8). (*p<0.05, **p<0.01). (TIF) [file ppat.1007569.s002.tif]

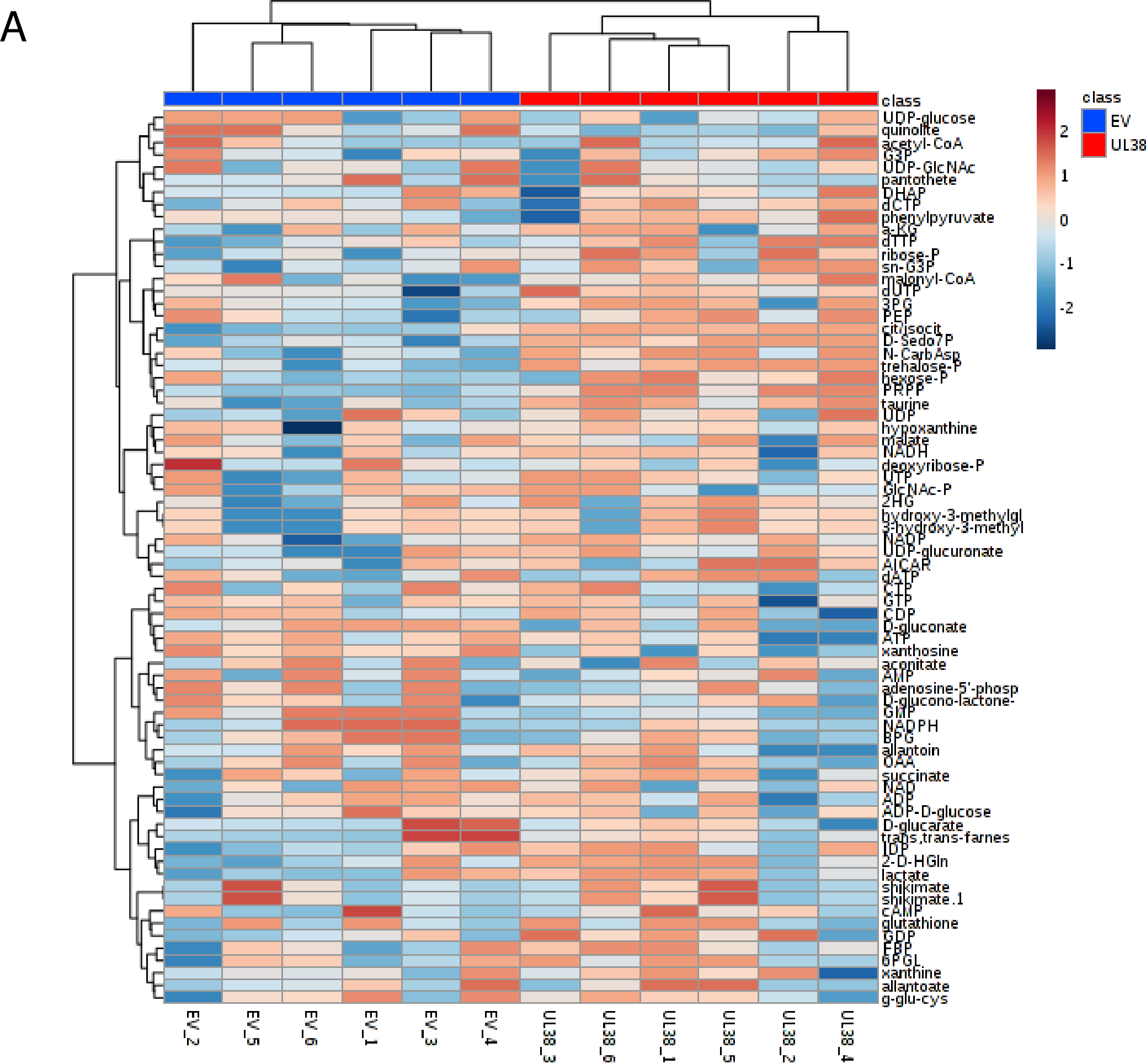

Supplement: S3 Fig — Confluent MRC5 cells expressing an empty vector control (EV) or UL38 protein (UL38) were cultured in serum free media for 24h. Cells were then quenched and extracted for analysis. Absolute intracellular metabolite concentrations were determined by LC-MS/MS and normalized to protein levels. (A) Heatmap of clustered metabolite pools. Values are means ± SE (n = 6). (*p<0.05, **p<0.01). (TIF) [file ppat.1007569.s003.tif]

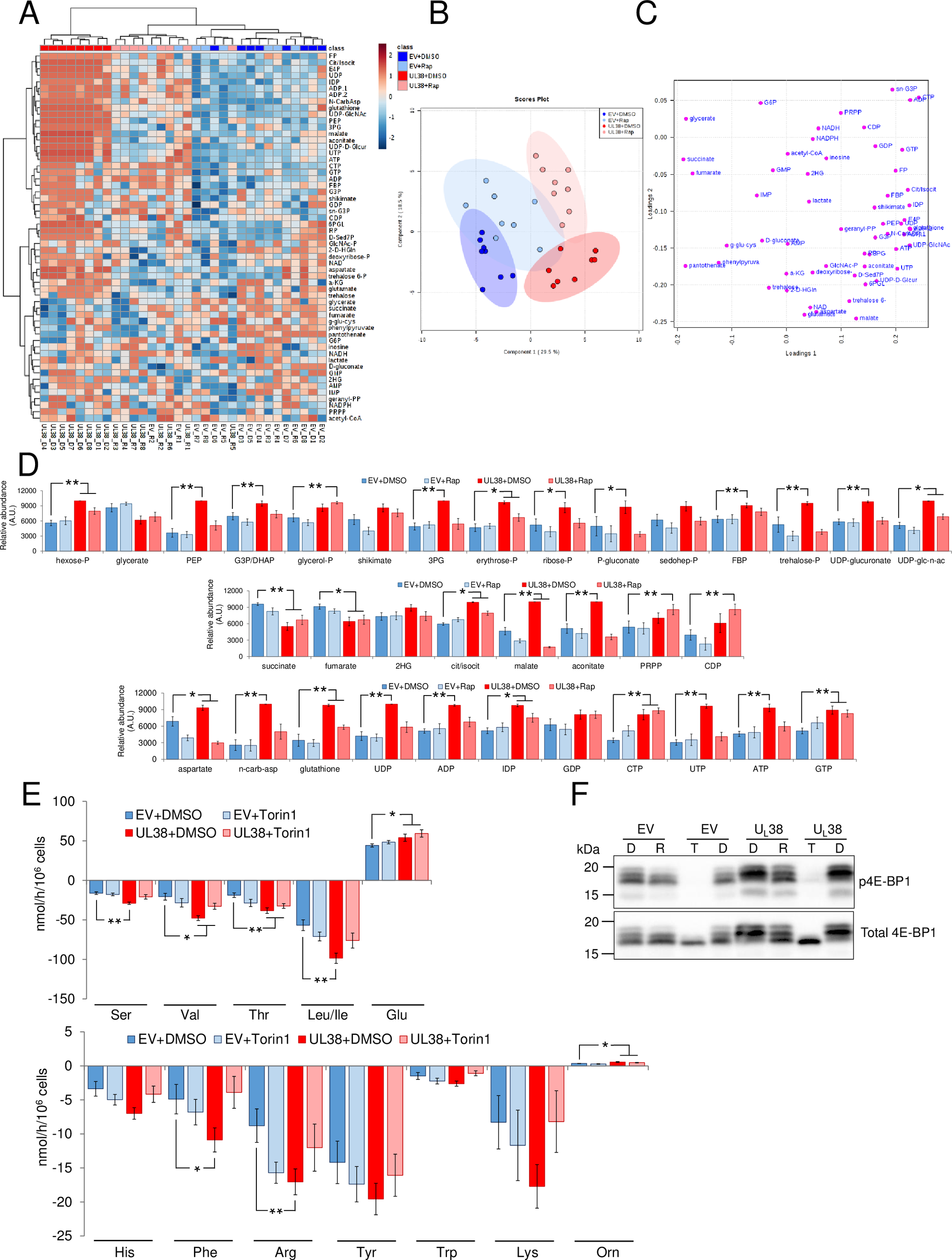

Supplement: S4 Fig — (A-D) Confluent MRC5 cells expressing an empty vector control (EV) or UL38 protein (UL38) were cultured in serum free media containing DMSO (+DMSO) or 100 nm of rapamycin (+Rap) for 24h. Cells were then quenched and extracted. Absolute intracellular metabolite concentrations were determined by LC-MS/MS and normalized to protein levels. (A) Heatmap of clustered metabolite pools. (B) Partial least-squares discriminant analysis (PLS-DA) of metabolic concentrations. (C) Loading plot for PLS-DA model. (D) Plotted selected metabolites. Values are means ± SE (n = 8). (E) Confluent MRC5 cells expressing EV or UL38 protein were cultured for 24h in serum free media containing DMSO (+DMSO) or Torin-1 (+Torin1). Conditioned medium and cells were harvested after 24h for analysis. Values are means ± SE. (n = 8) (*p<0.05, **p<0.01). (F) Western blot analysis of drug treated EV and UL38 cells (D = DMSO; R = Rapamycin; T = Torin1). Samples correspond to experiments described in Fig 4. (TIF) [file ppat.1007569.s004.tif]

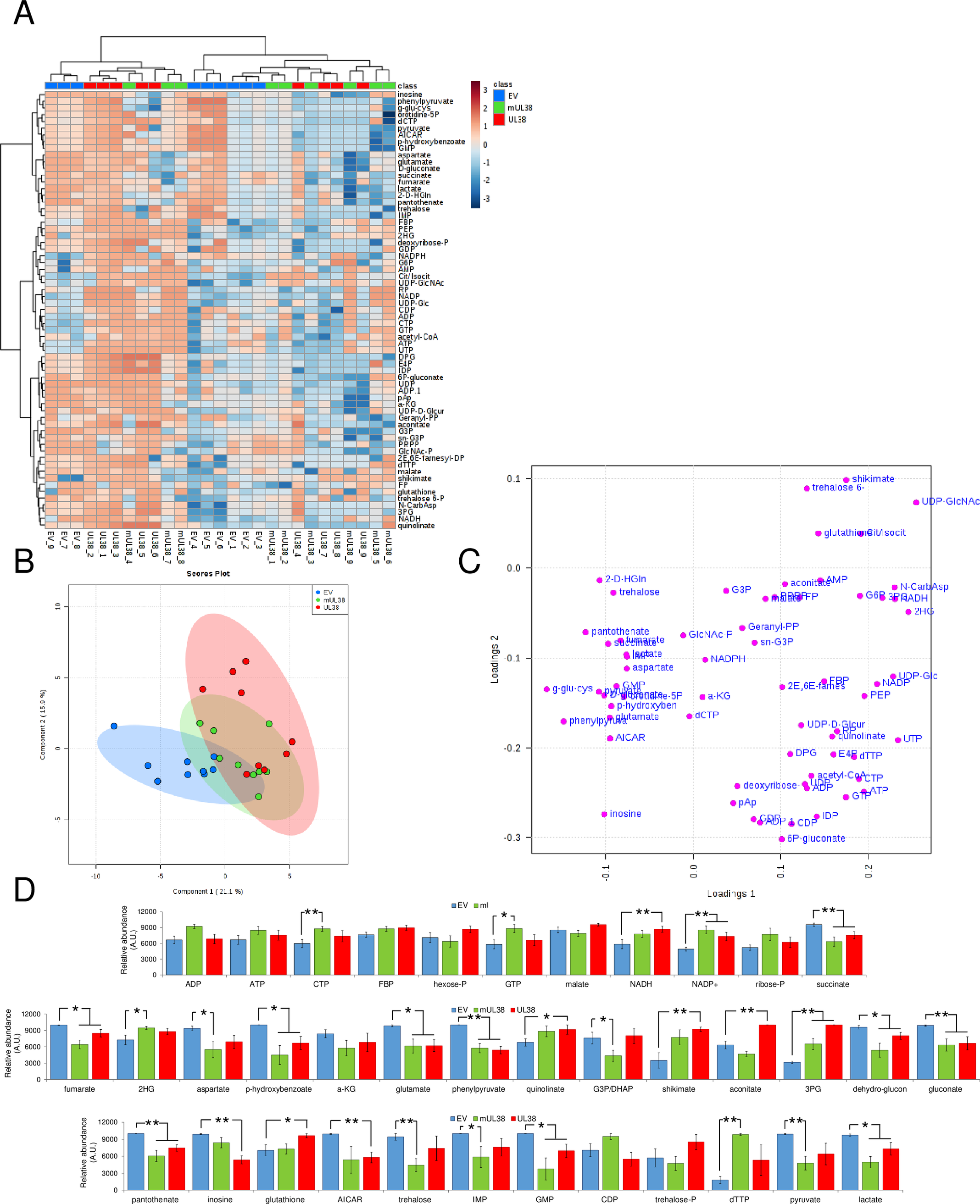

Supplement: S5 Fig — Confluent MRC5 cells expressing an empty vector control (EV), mutant UL38 T23A/Q24A (mUL38) or WT UL38 (UL38) were cultured in serum free media for 24h prior to metabolic quenching and extraction. Cellular absolute intracellular metabolite concentrations were determined by LC-MS/MS and normalized to protein levels. (A) Heatmap of clustered metabolite pools. (B) Partial least-squares discriminant analysis (PLS-DA) of metabolic concentrations. (C) Loading plot for PLS-DA model. (D) Plotted selected metabolites. Values are means ± SE (n = 9). (*p<0.05, **p<0.01). (TIF) [file ppat.1007569.s005.tif]

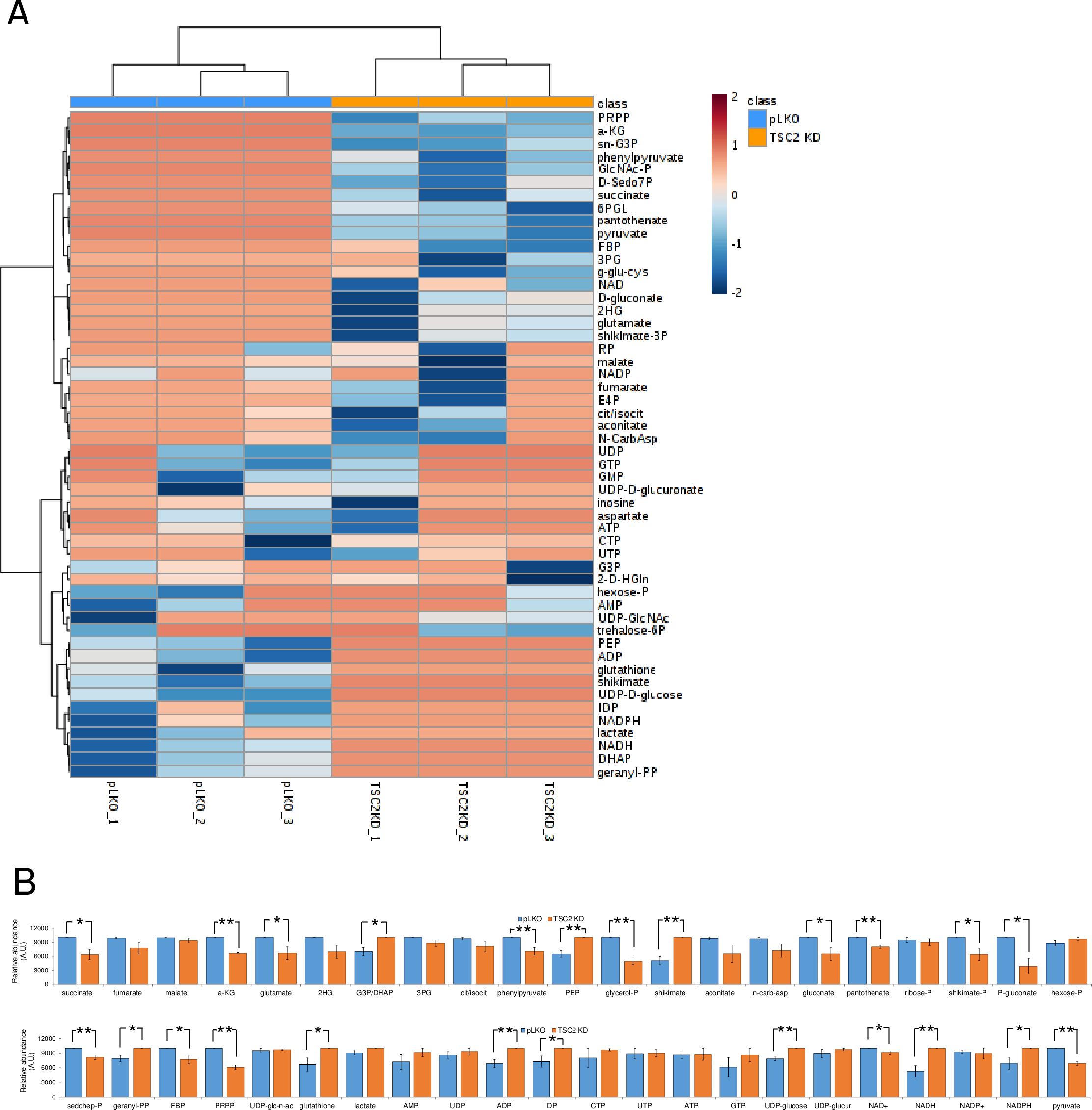

Supplement: S6 Fig — HFF cells were transduced with control (pLKO) or TSC2-specific shRNA (TSC2 KD)-expressing lentiviruses and selected. Confluent cells were cultured in serum free media for 24h before quenching and extraction. Absolute intracellular metabolite concentrations were determined by LC-MS/MS and normalized to protein levels. (A) Heatmap of clustered metabolite pools. (B) Plotted selected metabolites. Values are means ± SE (n = 3). (TIF) [file ppat.1007569.s006.tif]
